# Supplementary material for: Unsupervised machine learning reveals risk stratifying glioblastoma tumor cells
Source: eLife. 2020 Jun 23;9:e56879. doi: 10.7554/eLife.56879 (PMC7340505; doi:10.7554/eLife.56879)
Supplement: Supplementary file 1. [file elife-56879-supp1.docx]

**Supplemental Table 1. Comparison of Citrus and RAPID**

|  | **Citrus** | **RAPID** |
| --- | --- | --- |
| Finding cell clusters | Unsupervised  (hierarchical clustering, cells may be in > 1 cluster) | Unsupervised  (various: FlowSOM*, dbSCAN, KNN) |
| Determining number of clusters to seek | Unsupervised  (must be >5% of sample) | Unsupervised  (automatically chosen based on prioritizing low intra-cluster variance) |
| Modeling cluster features | Supervised, multivariate  (lasso regularized logistic regression, nearest shrunken centroid) | Unsupervised, univariate  (median or MEM statistical description of cluster) |
| Splitting patients into groups | Supervised, prior to clustering  (expert assigns patients to groups) | Unsupervised, post-clustering  (cluster abundance determines cut points, tested with a Cox model of hazards) |

*used in the work described above
