# Supplementary material for: Unsupervised machine learning reveals risk stratifying glioblastoma tumor cells
Source: eLife. 2020 Jun 23;9:e56879. doi: 10.7554/eLife.56879 (PMC7340505; doi:10.7554/eLife.56879)
Supplement: Supplementary file 3. [file elife-56879-supp3.docx]

| **Sample ID**  **Supplementary Table 3 – Glioblastoma Patient Characteristics** | **Gender** | **Age** | **TMZ** | **RT** | **EOR** | **MGMT meth** | **Stable GNP Total** | **Stable GPP Total** | **PFS (days)** | **PFS Status** | **OS (days)** | **OS Status** |
| --- | --- | --- | --- | --- | --- | --- | --- | --- | --- | --- | --- | --- |
| LC06 | Male | 41 | Yes | Yes | GTR | No | 0.0 | 91.1 | 1539 | Censored | 1588 | Alive |
| W11 | Male | 69 | Yes | Yes | GTR | No | 0.2 | 58.7 | 491 | Progressed | 896 | Dead |
| LC26 | Male | 71 | Yes | Yes | STR | Yes | 0.2 | 21.6 | 363 | Progressed | 836 | Alive |
| RT14 | Female | 55 | Yes | Yes | GTR | Yes | 0.6 | 19.3 | 472 | Progressed | 571 | Dead |
| K01 | Female | 59 | Yes | Yes | STR | No | 0.0 | 14.8 | 176 | Progressed | 364 | Dead |
| LC04 | Male | 65 | Yes | Yes | GTR | No | 0.2 | 14.2 | 263 | Progressed | 918 | Dead |
| LC25 | Female | 60 | Yes | Yes | GTR | No | 2.3 | 10.1 | 185 | Progressed | 731 | Dead |
| W02 | Male | 75 | Yes | Yes | STR | Yes | 1.8 | 8.3 | 446 | Progressed | 507 | Dead |
| W05 | Male | 60 | Yes | Yes | STR | No | 0.2 | 4.2 | 241 | Progressed | 282 | Dead |
| LC22 | Male | 64 | Yes | Yes | STR | No | 0.3 | 4.2 | 263 | Progressed | 366 | Dead |
| W12 | Male | 50 | Yes | Yes | STR | Yes | 2.4 | 4.5 | 190 | Progressed | 723 | Dead |
| W14 | Male | 47 | Yes | Yes | STR | Yes | 0.1 | 1.5 | 125 | Progressed | 488 | Dead |
| LC27 | Male | 62 | Yes | Yes | STR | No | 0.2 | 2.1 | 79 | Progressed | 252 | Dead |
| RT01 | Female | 69 | Yes | Yes | STR | No | 0.0 | 1.0 | 162 | Progressed | 198 | Dead |
| RT10 | Male | 56 | No | No | STR | Yes | 0.2 | 0.7 | 22 | Progressed | 113 | Dead |
| LC21 | Female | 69 | Yes | Yes | GTR | Yes | 0.9 | 2.5 | 267 | Progressed | 267 | Dead |
| W15 | Male | 50 | Yes | Yes | STR | Yes | 0.4 | 0.4 | 603 | Progressed | 697 | Dead |
| RT15 | Female | 80 | No | Yes | STR | No | 0.8 | 1.0 | 98 | Progressed | 353 | Dead |
| LC08 | Female | 55 | Yes | Yes | STR | No | 1.5 | 0.0 | 210 | Progressed | 441 | Dead |
| LC11 | Female | 76 | Yes | Yes | STR | Yes | 2.1 | 0.3 | 111 | Progressed | 411 | Dead |
| LC18 | Male | 69 | Yes | Yes | STR | No | 3.2 | 0.3 | 133 | Progressed | 240 | Dead |
| W04 | Male | 60 | Yes | Yes | STR | No | 3.2 | 0.1 | 220 | Progressed | 456 | Dead |
| LC03 | Male | 60 | No | No | STR | No | 5.6 | 0.1 | 57 | Progressed | 57 | Dead |
| LC09 | Male | 68 | Yes | Yes | STR | Yes | 4.6 | 0.0 | 66 | Progressed | 110 | Dead |
| LC10 | Male | 78 | No | Yes | STR | No | 7.8 | 0.0 | 117 | Censored | 178 | Dead |
| RT07 | Female | 70 | Yes | Yes | STR | No | 38.1 | 0.1 | 49 | Progressed | 57 | Dead |
| LC12 | Male | 40 | Yes | Yes | GTR | Yes | 55.4 | 0.1 | 606 | Progressed | 710 | Dead |
| LC02 | Male | 67 | No | No | STR | No | 80.1 | 0.0 | 53 | Progressed | 53 | Dead |

| TMZ = temozolomide  RT = radiation | EOR = extent of resection  STR = subtotal resection | GTR = gross total resection  MGMT meth = MGMT promoter methylation |
| --- | --- | --- |
