# Supplementary material for: Unsupervised machine learning reveals risk stratifying glioblastoma tumor cells
Source: eLife. 2020 Jun 23;9:e56879. doi: 10.7554/eLife.56879 (PMC7340505; doi:10.7554/eLife.56879)
Supplement: Supplementary file 4. [file elife-56879-supp4.docx]

**Supplementary Table 4 – Mass cytometry antibody panels**

| **Target** | **Mass** | **Clone** | **Signaling & proteins** | | **Stain** | | |
| --- | --- | --- | --- | --- | --- | --- | --- |
|  |  |  | **Panel** | **t-SNE** | **Live** | **Sap** | **MeOH** |
| Rhodium | 103 | - | ● |  | ✓ |  |  |
| Cyclin B1 | 139 | GNS-1 | ● |  |  |  | ✓ |
| TUJ1 | 141 | TUJ1 | ● | ⯀ |  |  | ✓ |
| cCasp3 | 142 | 5A1E | ● |  |  |  | ✓ |
| CD117 | 143 | 104D2 | ● | ⯀ | ✓ |  |  |
| S100B | 144 | 19/S100B | ● | ⯀ |  |  | ✓ |
| CD31 | 145 | WM59 | ● | ⯀* | ✓ |  |  |
| ɣH2AX | 147 | JBW301 | ● |  |  |  | ✓ |
| CD34 | 148 | 581 | ● | ⯀ | ✓ |  |  |
| p-4E-BP1 (T37/T46) | 149 | 236B4 | ● |  |  |  | ✓ |
| p-STAT5 (Y694) | 150 | 47 | ● | ⯀ |  |  | ✓ |
| BMX | 151 | 40/BMX | ● |  |  |  | ✓ |
| p-AKT (S473) | 152 | D9E | ● | ⯀ |  |  | ✓ |
| p-STAT1 (Y701) | 153 | 58D6 | ● | ⯀ |  |  | ✓ |
| CD45 | 154 | HI30 | ● | ⯀* | ✓ |  |  |
| NCAM/CD56 | 155 | HCD56 | ● | ⯀ | ✓ |  |  |
| p-p38 (T180/Y182) | 156 | D3F9 | ● | ⯀ |  |  | ✓ |
| p-STAT3 (Y705) | 158 | 4/P-STAT3 | ● | ⯀ |  |  | ✓ |
| ITGα6/CD49F | 159 | GoH3 | ● | ⯀ | ✓ |  |  |
| CD133 | 160 | AC133 | ● | ⯀ | ✓ |  |  |
| PDGFRα | 161 | 16A1 | ● | ⯀ | ✓ |  |  |
| SOX2 | 163 | O30-678 | ● | ⯀ |  | ✓ |  |
| SSEA-1/CD15 | 164 | W6D3 | ● | ⯀ | ✓ |  |  |
| EGFR | 165 | AY13 | ● | ⯀ | ✓ |  |  |
| p-NFκB p65 (S529) | 166 | K10-895.12.50 | ● | ⯀ |  |  | ✓ |
| L1CAM | 167 | 5G3 | ● | ⯀ | ✓ |  |  |
| Nestin | 168 | 10C2 | ● | ⯀ |  |  | ✓ |
| CD44 | 169 | BJ18 | ● | ⯀ | ✓ |  |  |
| GFAP | 170 | 1B4 | ● | ⯀ |  |  | ✓ |
| p-ERK1/2 (T202/Y204) | 171 | D13.14.4E | ● | ⯀ |  |  | ✓ |
| p-S6 (S235/S236) | 172 | N7-548 | ● | ⯀ |  |  | ✓ |
| SOX10 | 173 | A-2 | ● |  |  |  | ✓ |
| HLA-DR | 174 | L243 | ● | ⯀ | ✓ |  |  |
| p-HH3 | 175 | HTA28 | ● |  |  |  | ✓ |
| Histone H3 | 176 | D1H2 | ● |  |  |  | ✓ |

● = included in the panel

⯀ = included for generation of t-SNE map

* Excluded from t-SNE analyses of only glioblastoma cells

Live = live surface stain

Sap = 0.02% saponin stain

MeOH = stain after ice-cold methanol permeabilization
