# Supplementary material for: Unsupervised machine learning reveals risk stratifying glioblastoma tumor cells
Source: eLife. 2020 Jun 23;9:e56879. doi: 10.7554/eLife.56879 (PMC7340505; doi:10.7554/eLife.56879)
Supplement: Supplementary file 6. [file elife-56879-supp6.pdf]

Supplementary File 6

Each page is specific for one of the 28 patients included in the main body of the text. Across the top of each page there are four t-SNE plots. From left to right: contour of all 28 patients, density contour of one patient, flowSOM cluster coloring of one patient, flowSOM cluster coloring of all 28 patients. Overall survival (OS) and progression-free survival (PFS) are noted on the left. Main body of the page is dedicated to t-SNE heat plots depicting marker expression on a single patient sample. On the right, flowSOM clusters are listed and percent of patient cells falling into each cluster is noted.

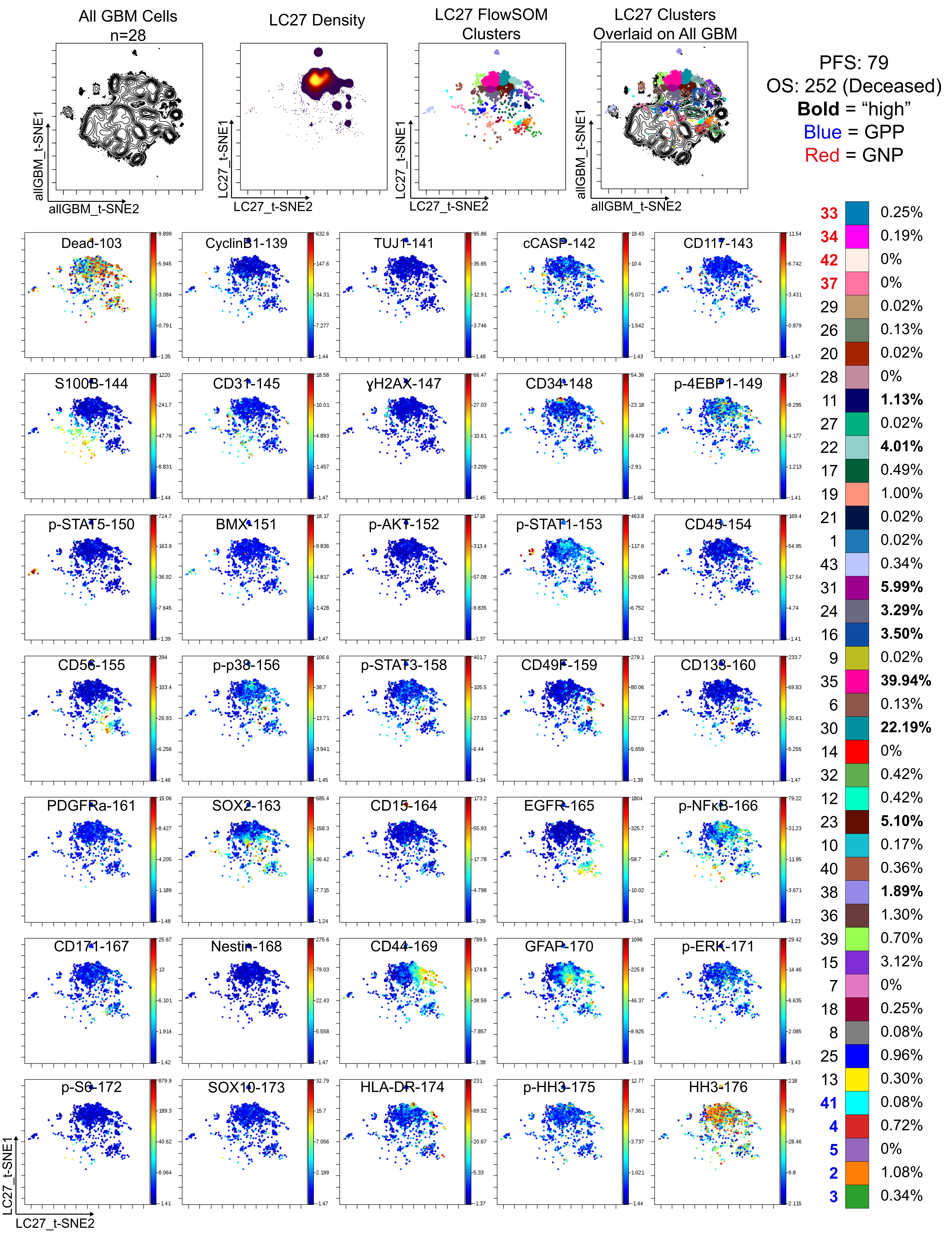
All GBM Cells
n=28
LC27 Density
LC27 FlowSOM Clusters
LC27 Clusters Overlaid on All GBM
allGBM_t-SNE1
allGBM_t-SNE2
LC27_t-SNE1
LC27_t-SNE2
PFS: 79
OS: 252 (Deceased)
Bold = "high"
Blue = GPP
Red = GNP
Dead-103
CyclinB1-139
TUJ1-141
cCASP-142
CD117-143
S100B-144
CD31-145
γH2AX-147
CD34-148
p-4EBP1-149
p-STAT5-150
BMX-151
p-AKT-152
p-STAT1-153
CD45-154
CD56-155
p-p38-156
p-STAT3-158
CD49F-159
CD133-160
PDGFRa-161
SOX2-163
CD15-164
EGFR-165
p-NFκB-166
CD171-167
Nestin-168
CD44-169
GFAP-170
p-ERK-171
p-S6-172
SOX10-173
HLA-DR-174
p-HH3-175
HH3-176
33 0.25%
34 0.19%
42 0%
37 0%
29 0.02%
26 0.13%
20 0.02%
28 0%
11 1.13%
27 0.02%
22 4.01%
17 0.49%
19 1.00%
21 0.02%
1 0.02%
43 0.34%
31 5.99%
24 3.29%
16 3.50%
9 0.02%
35 39.94%
6 0.13%
30 22.19%
14 0%
32 0.42%
12 0.42%
23 5.10%
10 0.17%
40 0.36%
38 1.89%
36 1.30%
39 0.70%
15 3.12%
7 0%
18 0.25%
8 0.08%
25 0.96%
13 0.30%
41 0.08%
4 0.72%
5 0%
2 1.08%
3 0.34%

Supplemental Single Cell Patient Information

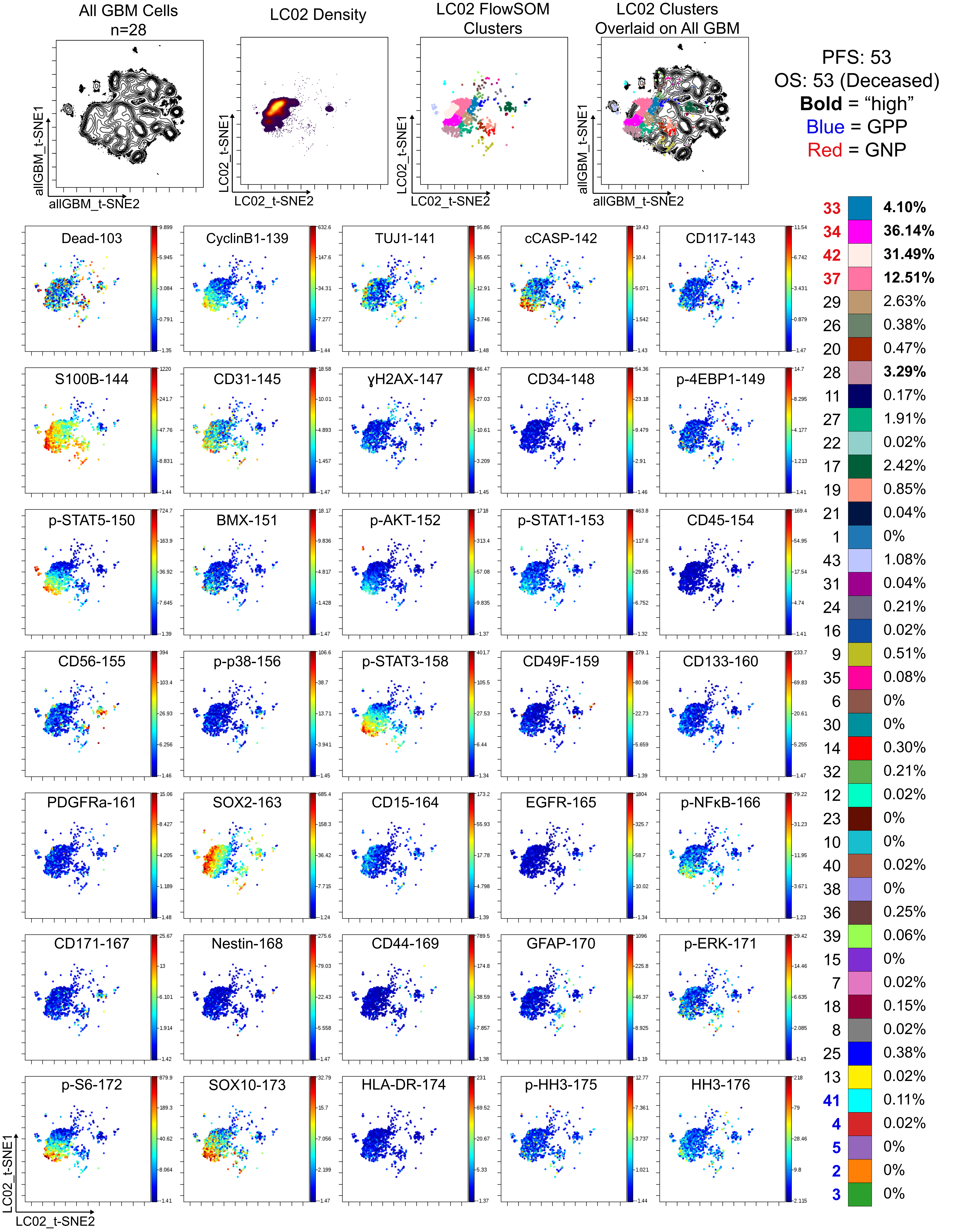

Supplemental Single Cell Patient Information

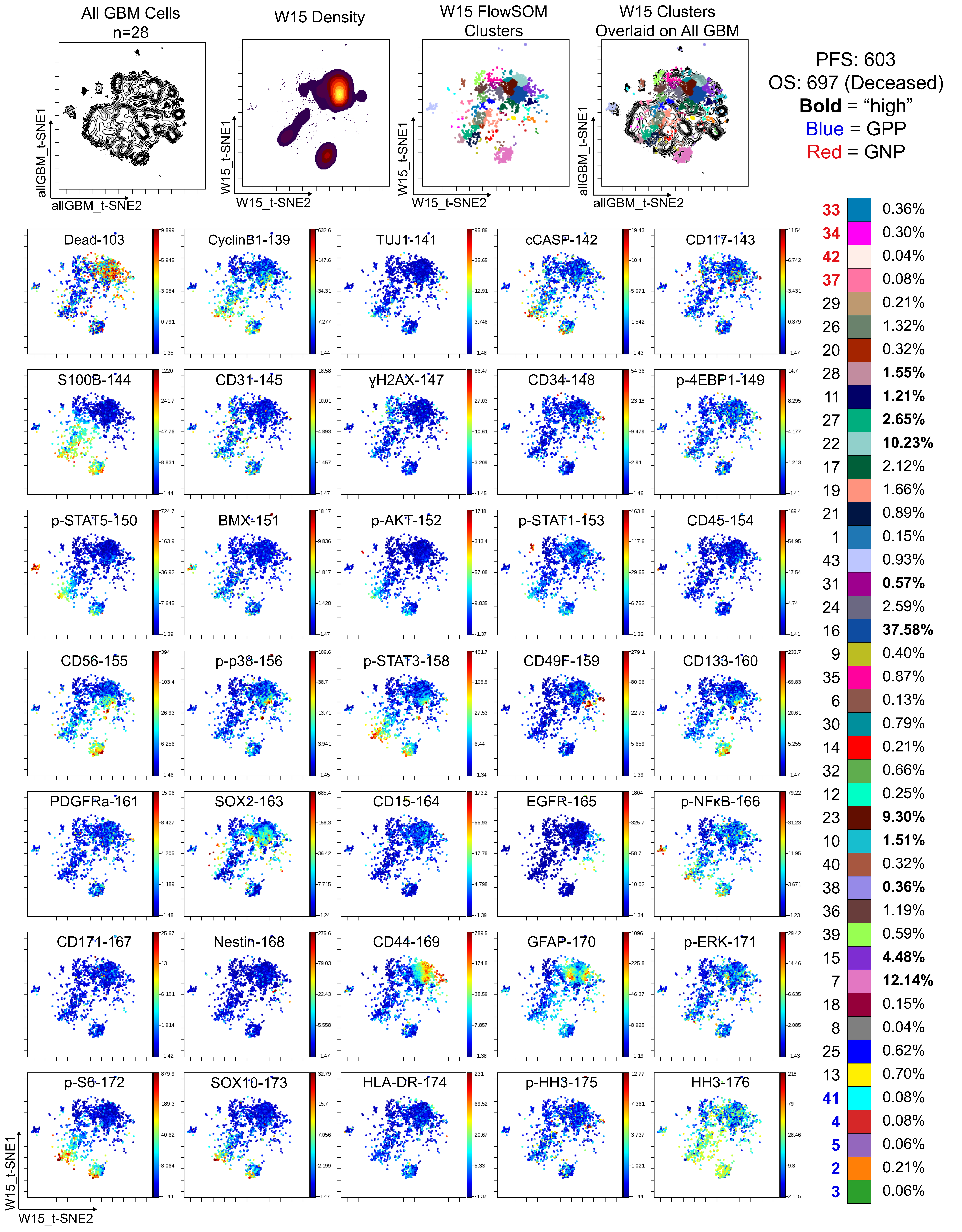

Supplemental Single Cell Patient Information

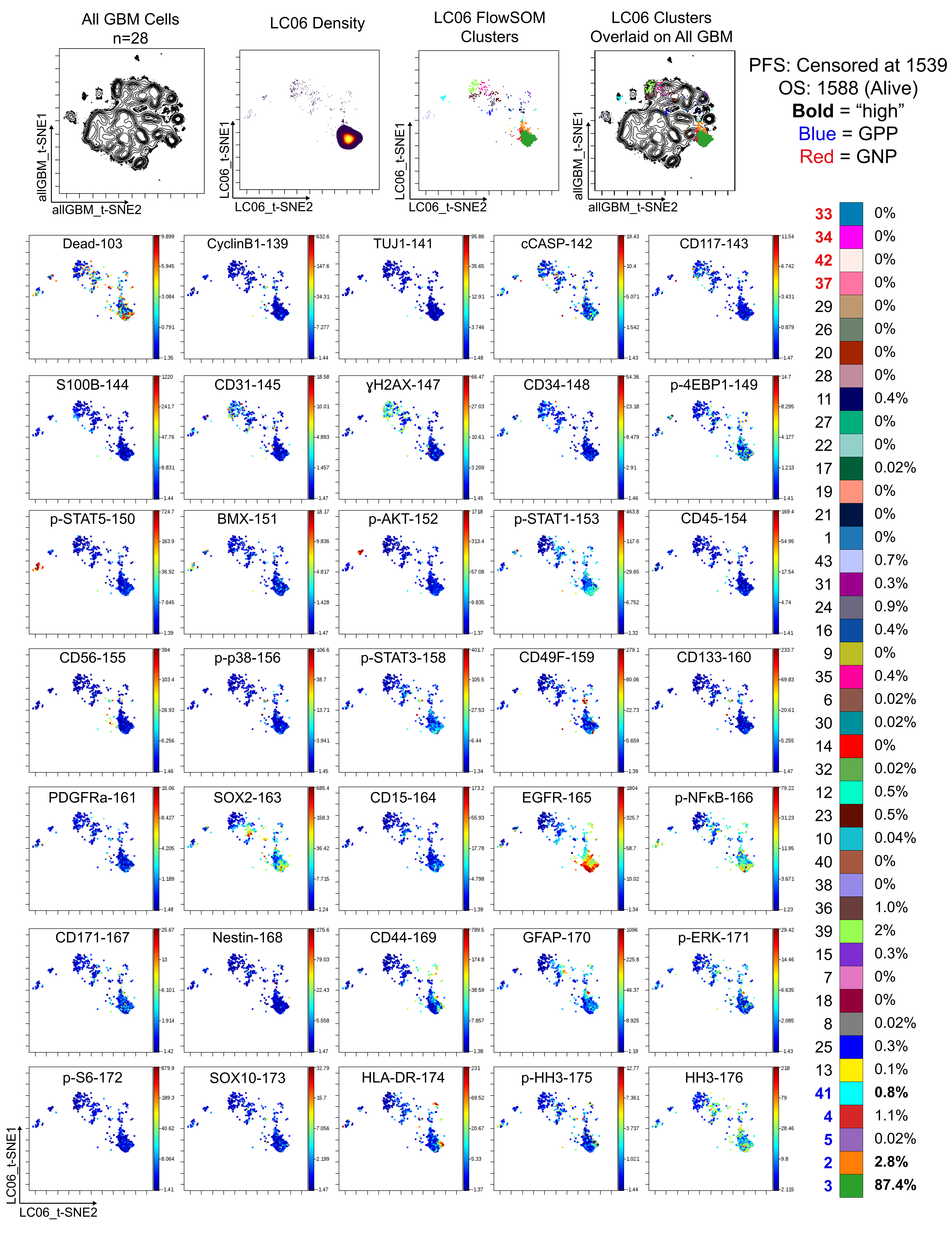

Supplemental Single Cell Patient Information

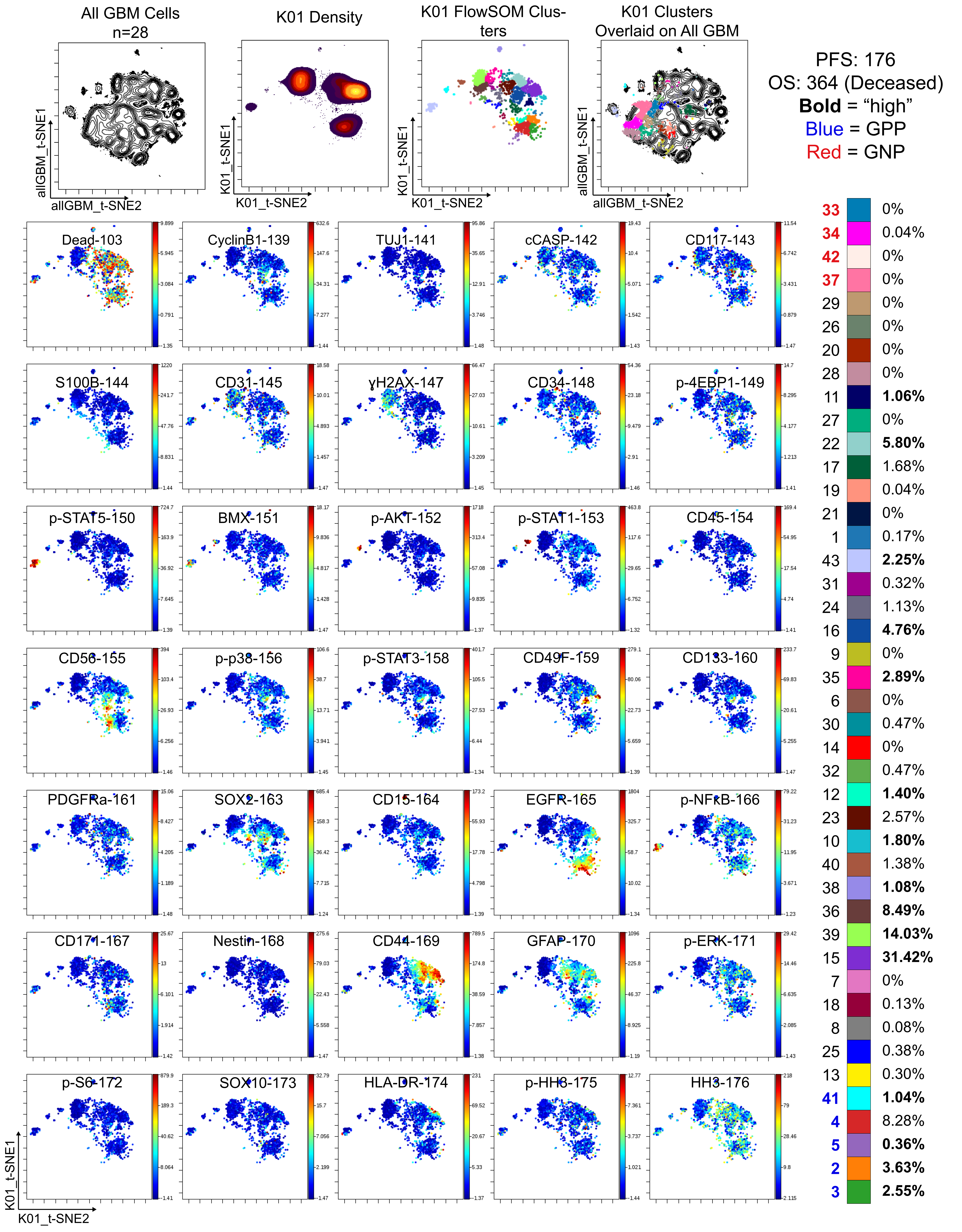

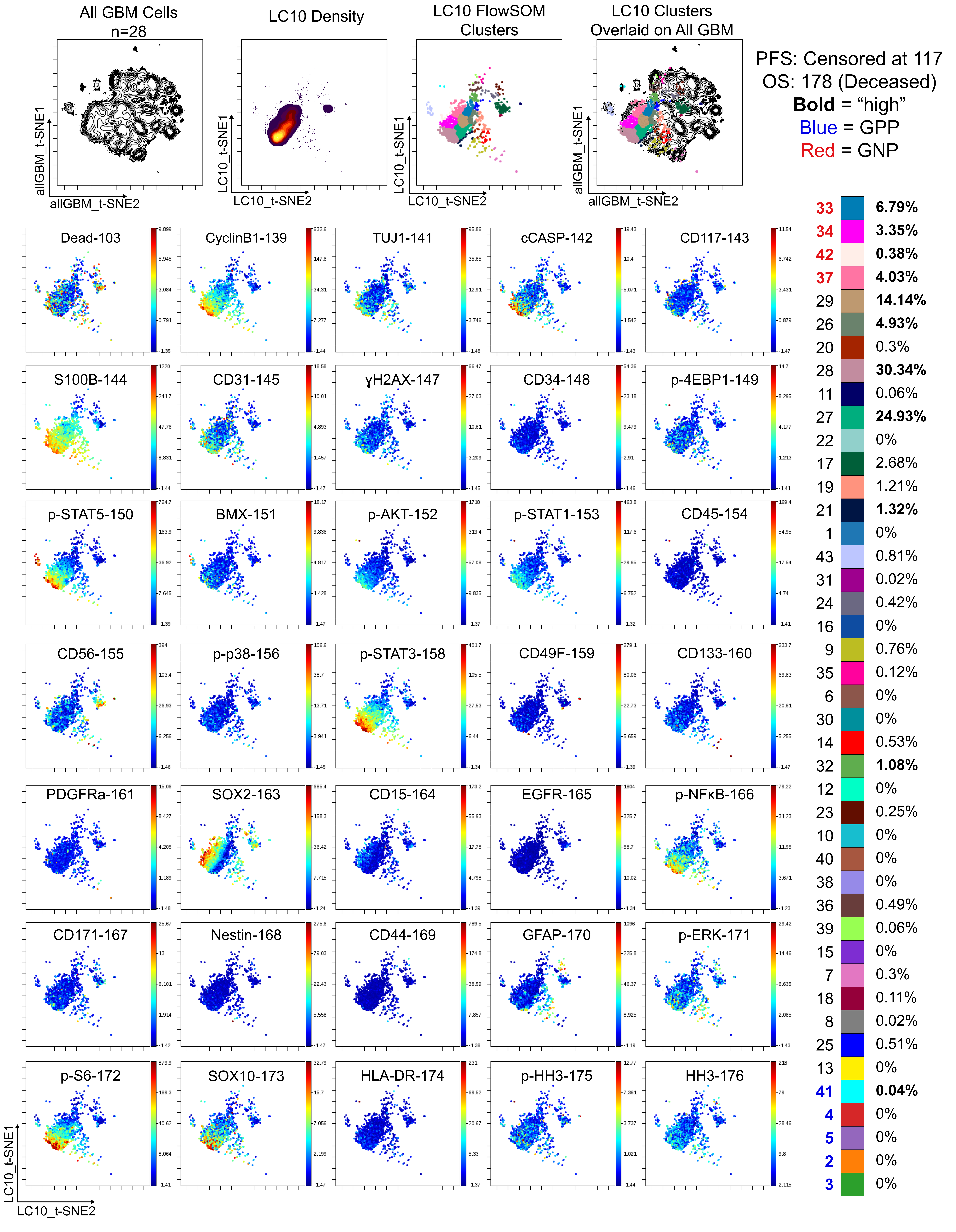
All GBM Cells n=28
LC10 Density
LC10 FlowSOM Clusters
LC10 Clusters Overlaid on All GBM
allGBM_t-SNE1
allGBM_t-SNE2
LC10_t-SNE1
LC10_t-SNE2
PFS: Censored at 117
OS: 178 (Deceased)
Bold = "high"
Blue = GPP
Red = GNP
33 6.79%
34 3.35%
42 0.38%
37 4.03%
29 14.14%
26 4.93%
20 0.3%
28 30.34%
11 0.06%
27 24.93%
22 0%
17 2.68%
19 1.21%
21 1.32%
1 0%
43 0.81%
31 0.02%
24 0.42%
16 0%
9 0.76%
35 0.12%
6 0%
30 0%
14 0.53%
32 1.08%
12 0%
23 0.25%
10 0%
40 0%
38 0%
36 0.49%
39 0.06%
15 0%
7 0.3%
18 0.11%
8 0.02%
25 0.51%
13 0%
41 0.04%
4 0%
5 0%
2 0%
3 0%
Dead-103
CyclinB1-139
TUJ1-141
cCASP-142
CD117-143
S100B-144
CD31-145
γH2AX-147
CD34-148
p-4EBP1-149
p-STAT5-150
BMX-151
p-AKT-152
p-STAT1-153
CD45-154
CD56-155
p-p38-156
p-STAT3-158
CD49F-159
CD133-160
PDGFRa-161
SOX2-163
CD15-164
EGFR-165
p-NFκB-166
CD171-167
Nestin-168
CD44-169
GFAP-170
p-ERK-171
p-S6-172
SOX10-173
HLA-DR-174
p-HH3-175
HH3-176

Supplemental Single Cell Patient Information

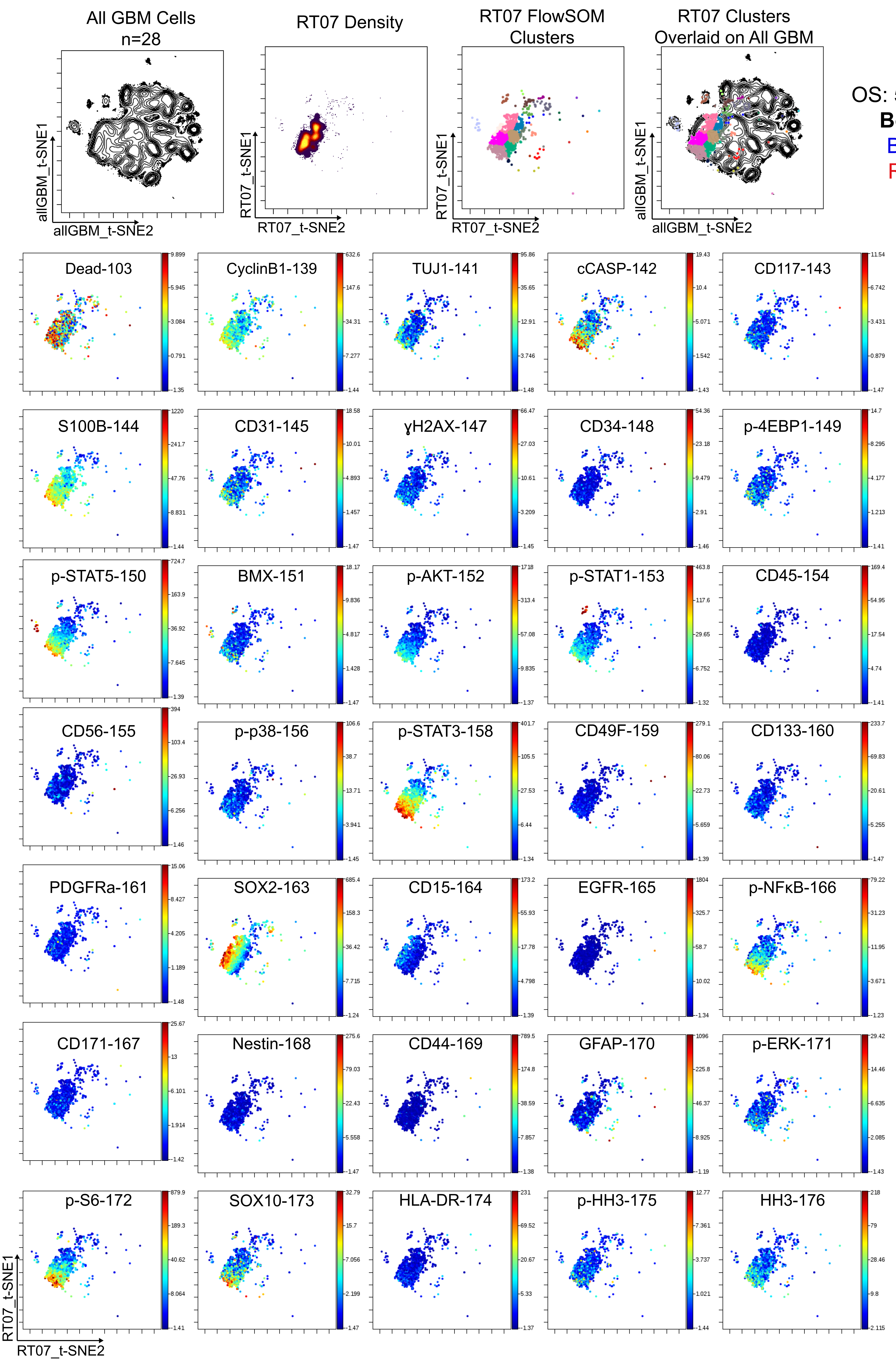

PFS: 49
OS: 57 (Deceased)
Bold = “high”
Blue = GPP
Red = GNP

**33**	**7.03%**
**37**	**13.10%**
**42**	**0.78%**
**34**	**24.20%**
29	**20.70%**
26	1.25%
20	0.02%
28	**17.64%**
11	0.02%
27	**11.23%**
22	0%
17	0.06%
19	0.23%
21	0.49%
1	0%
43	0.40%
31	0.11%
24	0.72%
16	0%
9	0.06%
35	0%
6	0%
30	0%
14	0.17%
32	0.40%
12	0%
23	0.02%
10	0.02%
40	0.64%
38	0%
36	0.42%
39	0.04%
15	0.02%
7	0.02%
18	0%
8	0.02%
25	0.11%
13	0%
**41**	0%
**4**	0.04%
**5**	0%
**2**	0.02%
**3**	0%

Supplemental Single Cell Patient Information

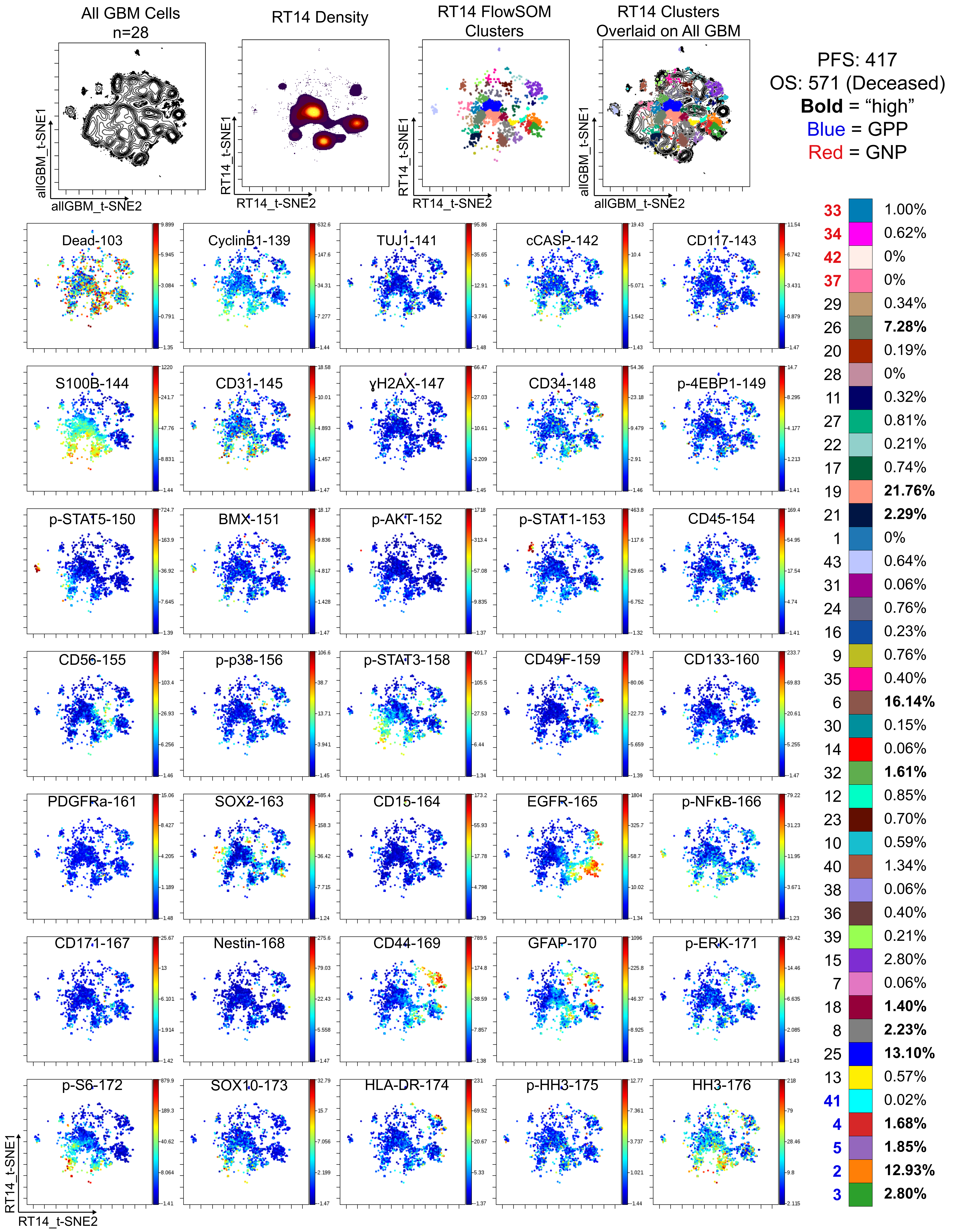

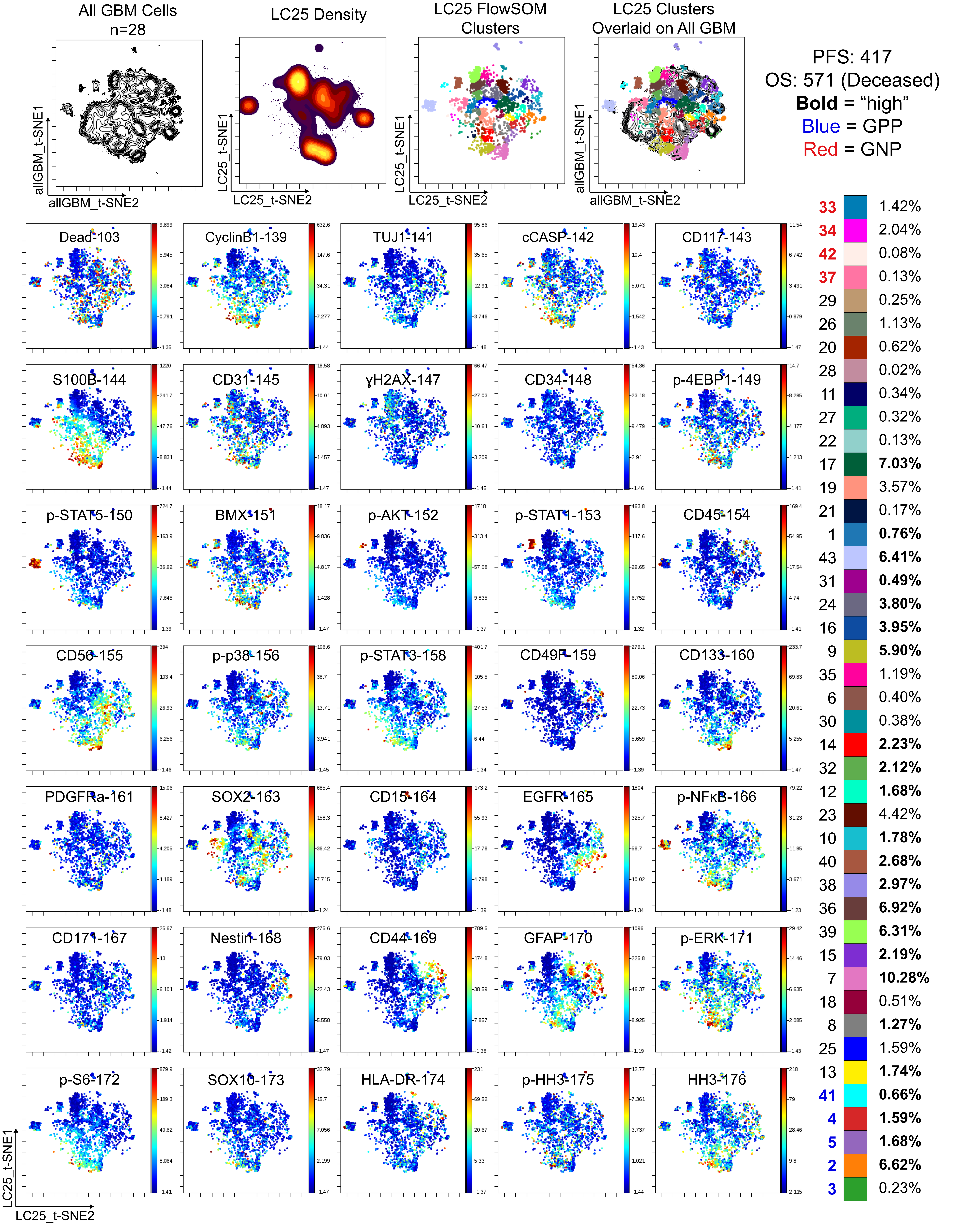
All GBM Cells
n=28
LC25 Density
LC25 FlowSOM Clusters
LC25 Clusters Overlaid on All GBM
allGBM_t-SNE1
allGBM_t-SNE2
LC25_t-SNE1
LC25_t-SNE2
PFS: 417
OS: 571 (Deceased)
Bold = "high"
Blue = GPP
Red = GNP
Dead-103
CyclinB1-139
TUJ1-141
cCASP-142
CD117-143
S100B-144
CD31-145
γH2AX-147
CD34-148
p-4EBP1-149
p-STAT5-150
BMX-151
p-AKT-152
p-STAT1-153
CD45-154
CD56-155
p-p38-156
p-STAT3-158
CD49F-159
CD133-160
PDGFRa-161
SOX2-163
CD15-164
EGFR-165
p-NFκB-166
CD171-167
Nestin-168
CD44-169
GFAP-170
p-ERK-171
p-S6-172
SOX10-173
HLA-DR-174
p-HH3-175
HH3-176
33 1.42%
34 2.04%
42 0.08%
37 0.13%
29 0.25%
26 1.13%
20 0.62%
28 0.02%
11 0.34%
27 0.32%
22 0.13%
17 7.03%
19 3.57%
21 0.17%
1 0.76%
43 6.41%
31 0.49%
24 3.80%
16 3.95%
9 5.90%
35 1.19%
6 0.40%
30 0.38%
14 2.23%
32 2.12%
12 1.68%
23 4.42%
10 1.78%
40 2.68%
38 2.97%
36 6.92%
39 6.31%
15 2.19%
7 10.28%
18 0.51%
8 1.27%
25 1.59%
13 1.74%
41 0.66%
4 1.59%
5 1.68%
2 6.62%
3 0.23%

Supplemental Single Cell Patient Information

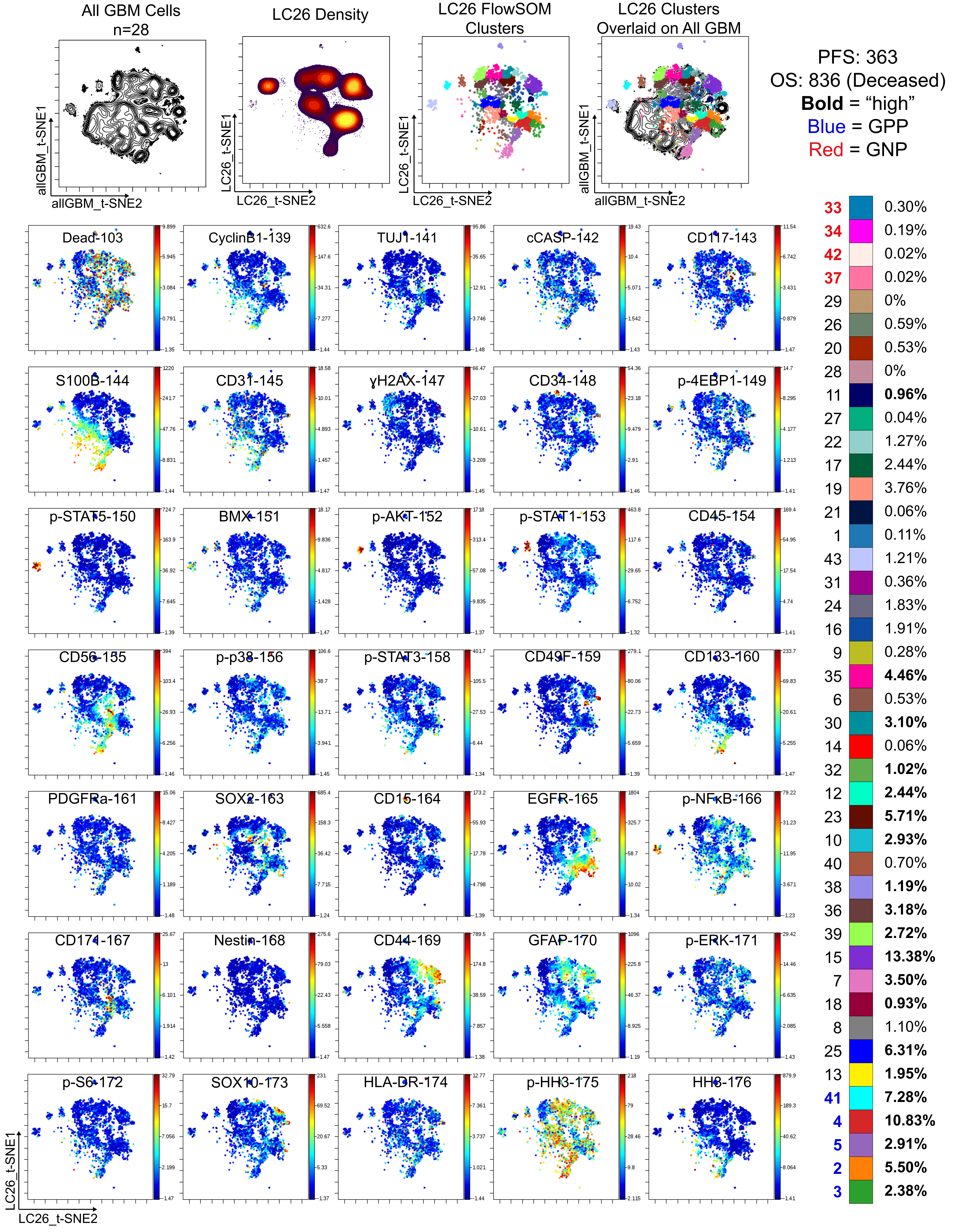

Supplemental Single Cell Patient Information

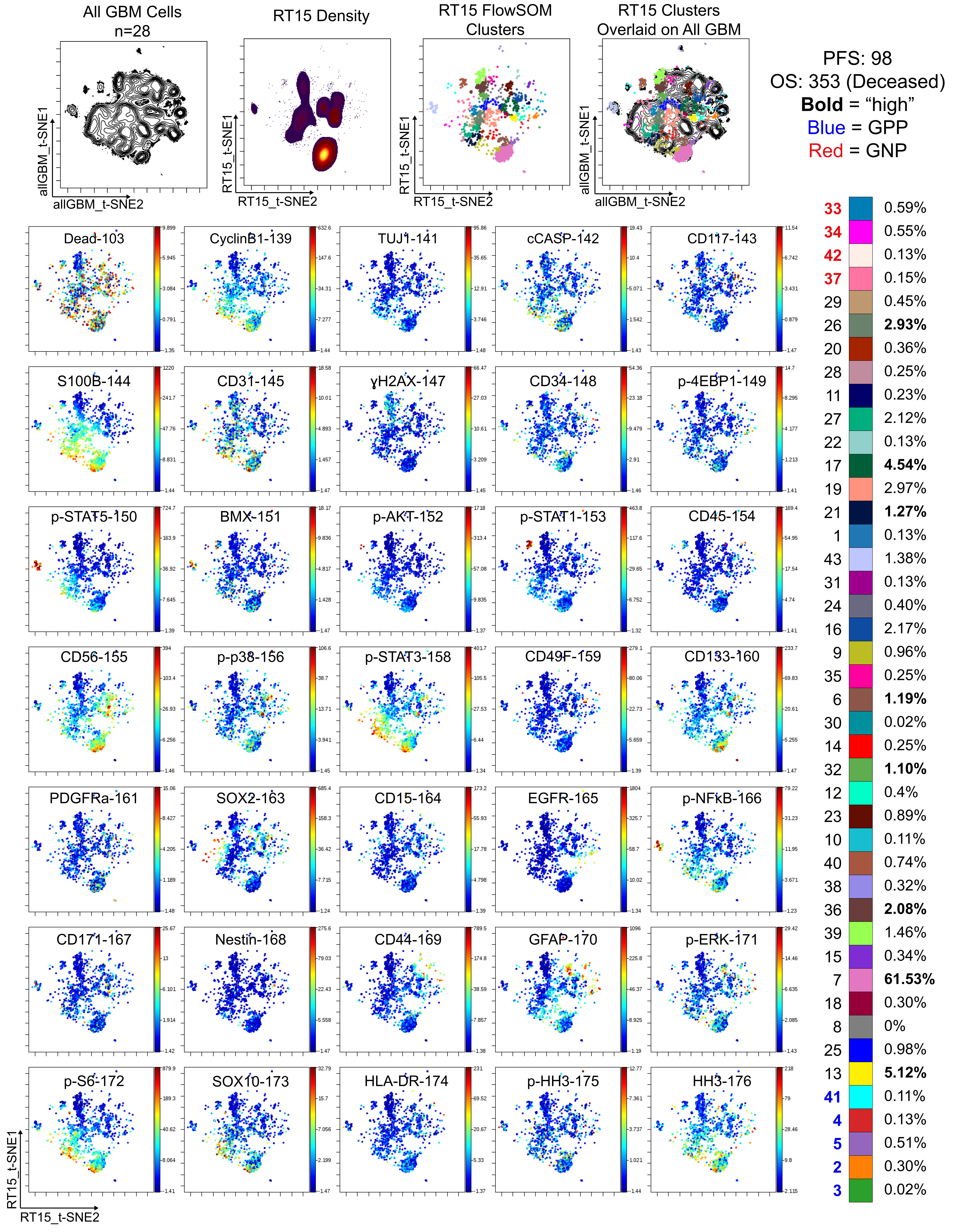

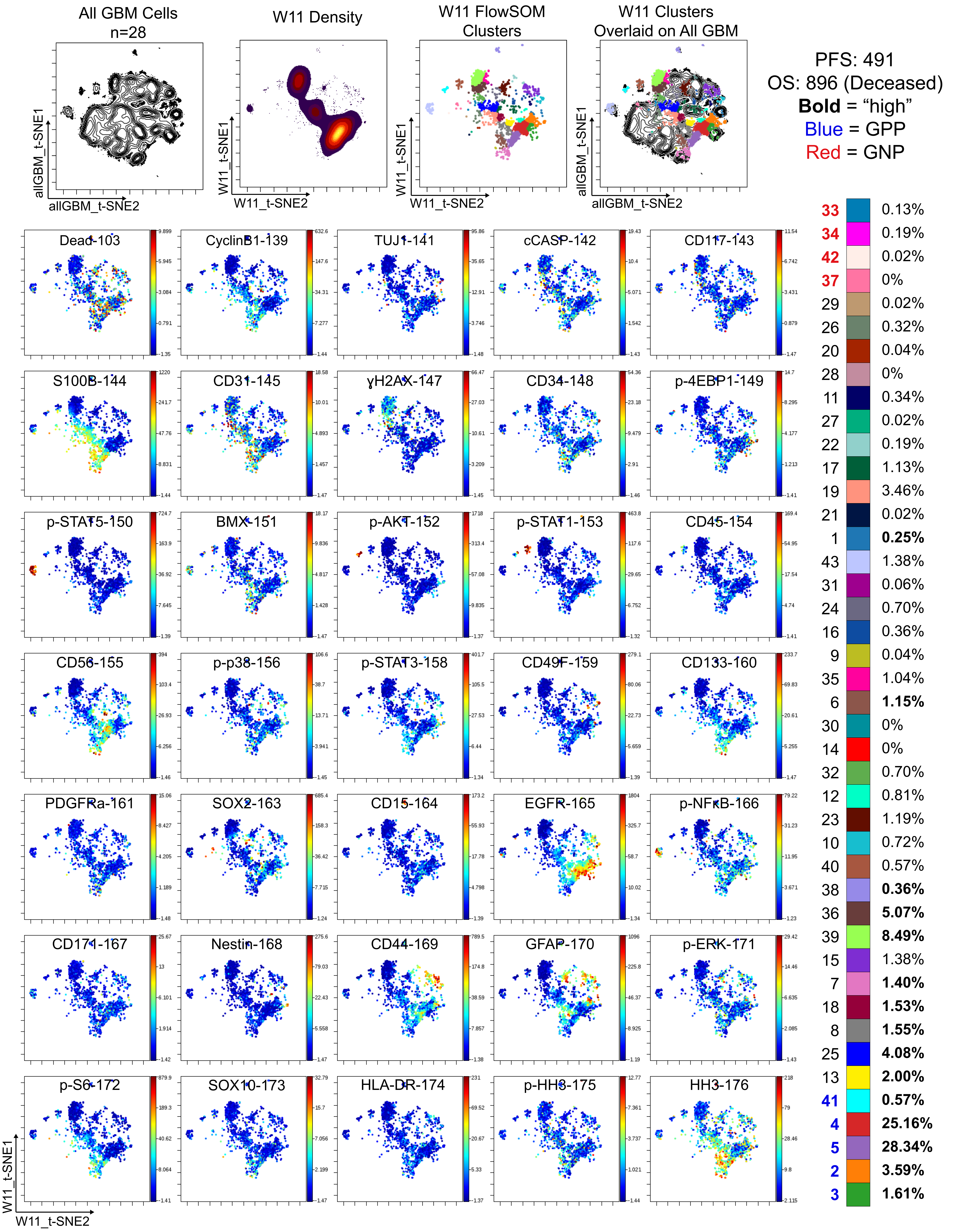
All GBM Cells
n=28
W11 Density
W11 FlowSOM Clusters
W11 Clusters Overlaid on All GBM
allGBM_t-SNE1
allGBM_t-SNE2
W11_t-SNE1
W11_t-SNE2
PFS: 491
OS: 896 (Deceased)
Bold = "high"
Blue = GPP
Red = GNP
33 0.13%
34 0.19%
42 0.02%
37 0%
29 0.02%
26 0.32%
20 0.04%
28 0%
11 0.34%
27 0.02%
22 0.19%
17 1.13%
19 3.46%
21 0.02%
1 0.25%
43 1.38%
31 0.06%
24 0.70%
16 0.36%
9 0.04%
35 1.04%
6 1.15%
30 0%
14 0%
32 0.70%
12 0.81%
23 1.19%
10 0.72%
40 0.57%
38 0.36%
36 5.07%
39 8.49%
15 1.38%
7 1.40%
18 1.53%
8 1.55%
25 4.08%
13 2.00%
41 0.57%
4 25.16%
5 28.34%
2 3.59%
3 1.61%
Dead-103
CyclinB1-139
TUJ1-141
cCASP-142
CD117-143
S100B-144
CD31-145
γH2AX-147
CD34-148
p-4EBP1-149
p-STAT5-150
BMX-151
p-AKT-152
p-STAT1-153
CD45-154
CD56-155
p-p38-156
p-STAT3-158
CD49F-159
CD133-160
PDGFRa-161
SOX2-163
CD15-164
EGFR-165
p-NFkB-166
CD171-167
Nestin-168
CD44-169
GFAP-170
p-ERK-171
p-S6-172
SOX10-173
HLA-DR-174
p-HH3-175
HH3-176

Supplemental Single Cell Patient Information

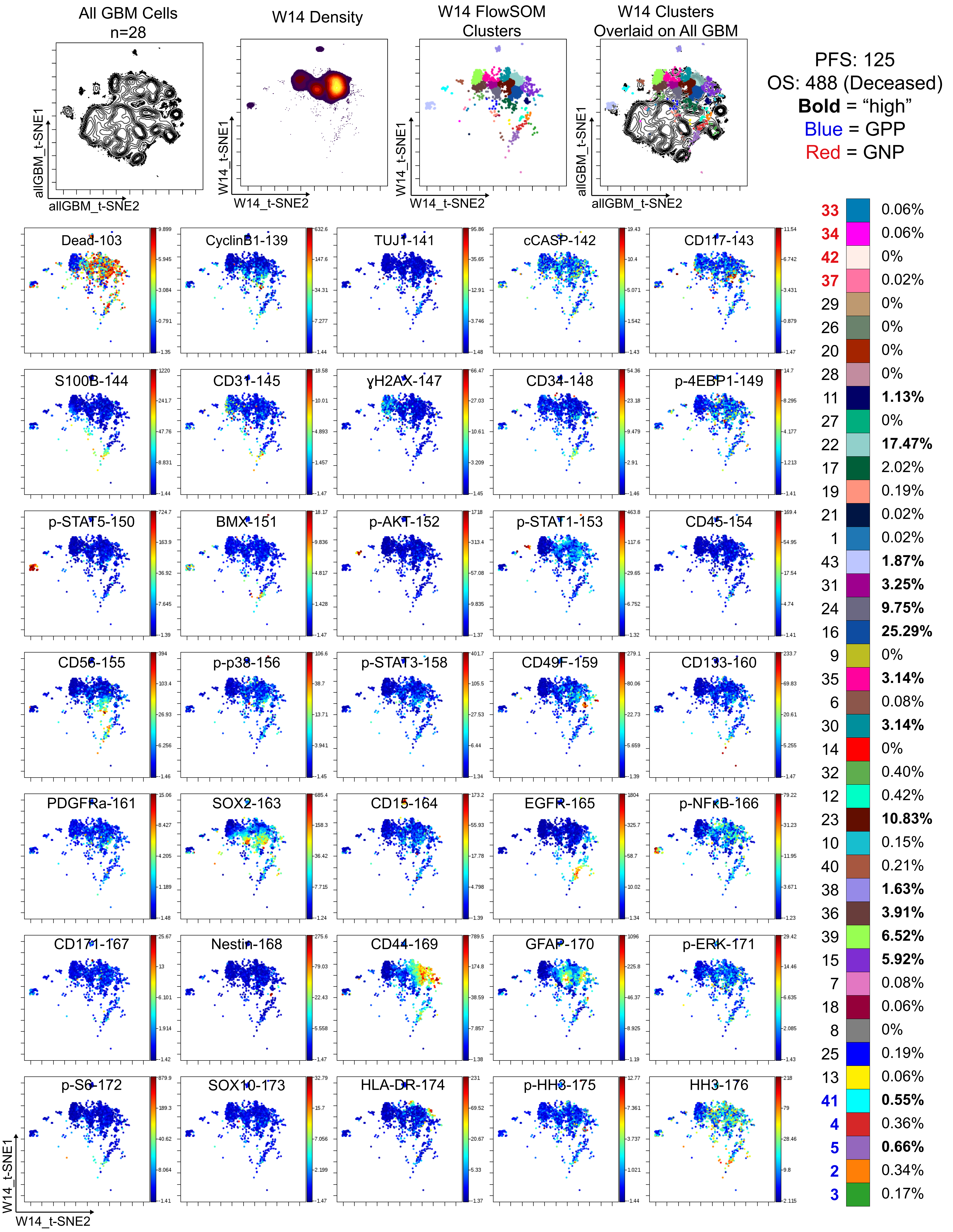

Supplemental Single Cell Patient Information

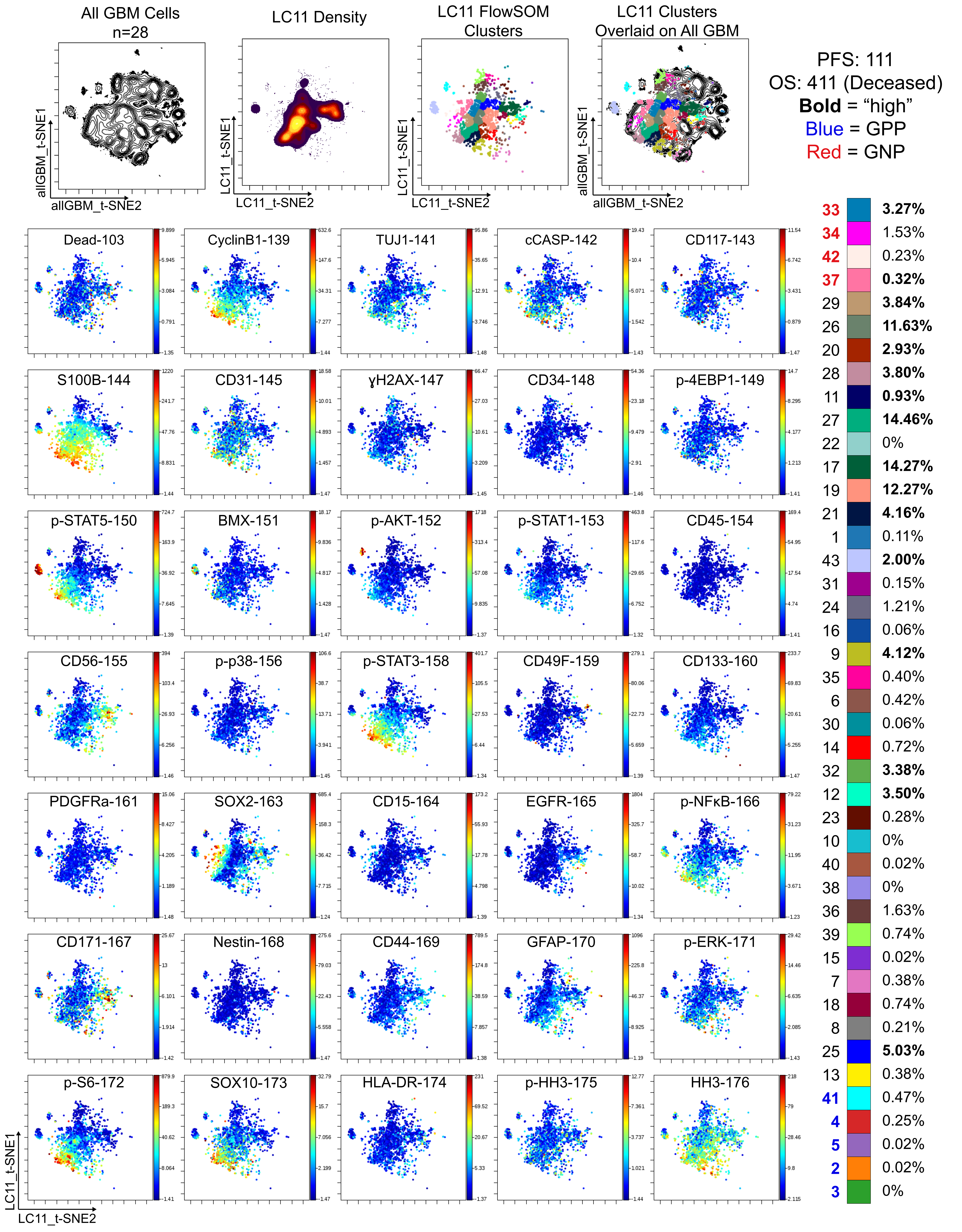

Supplemental Single Cell Patient Information

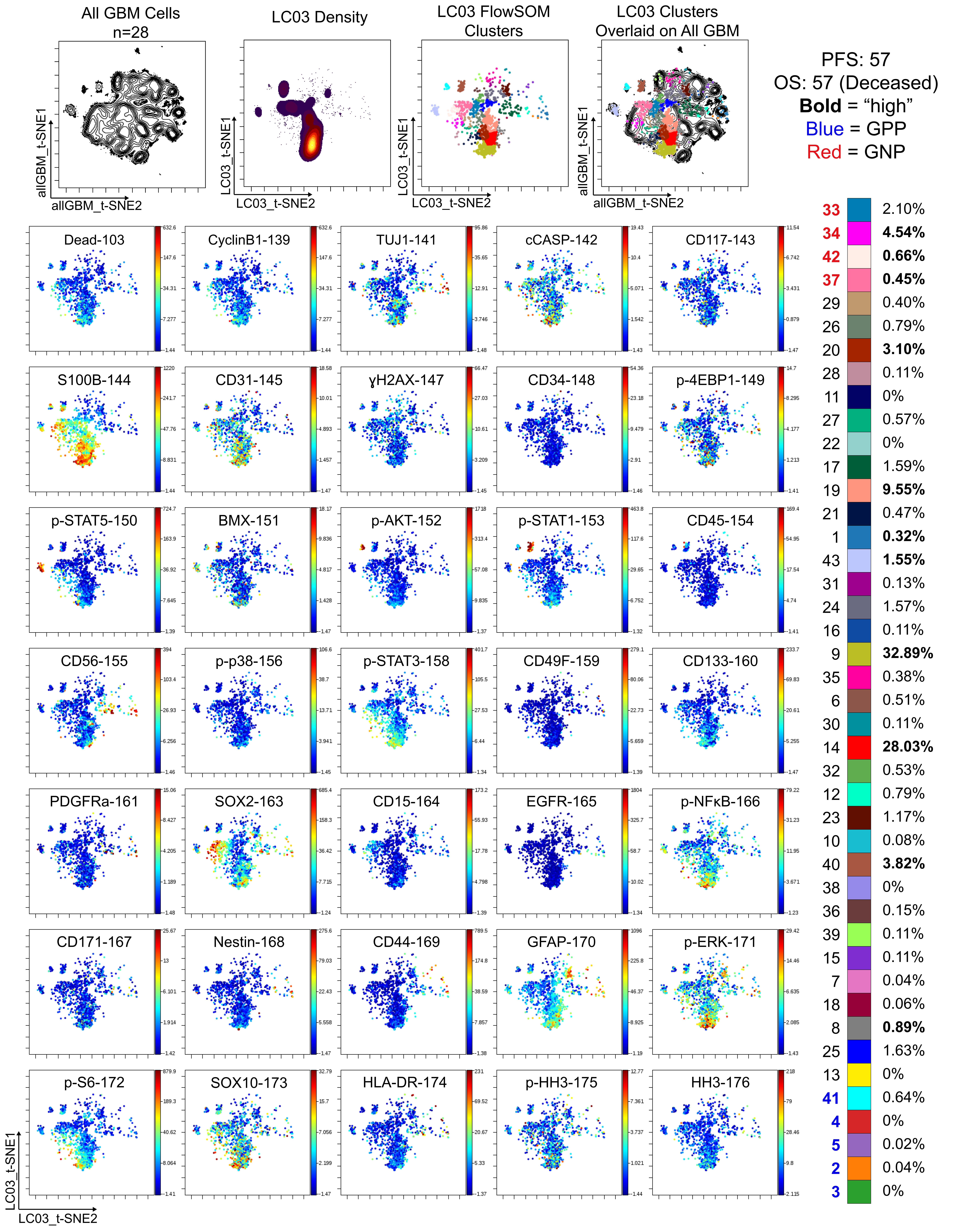

Supplemental Single Cell Patient Information

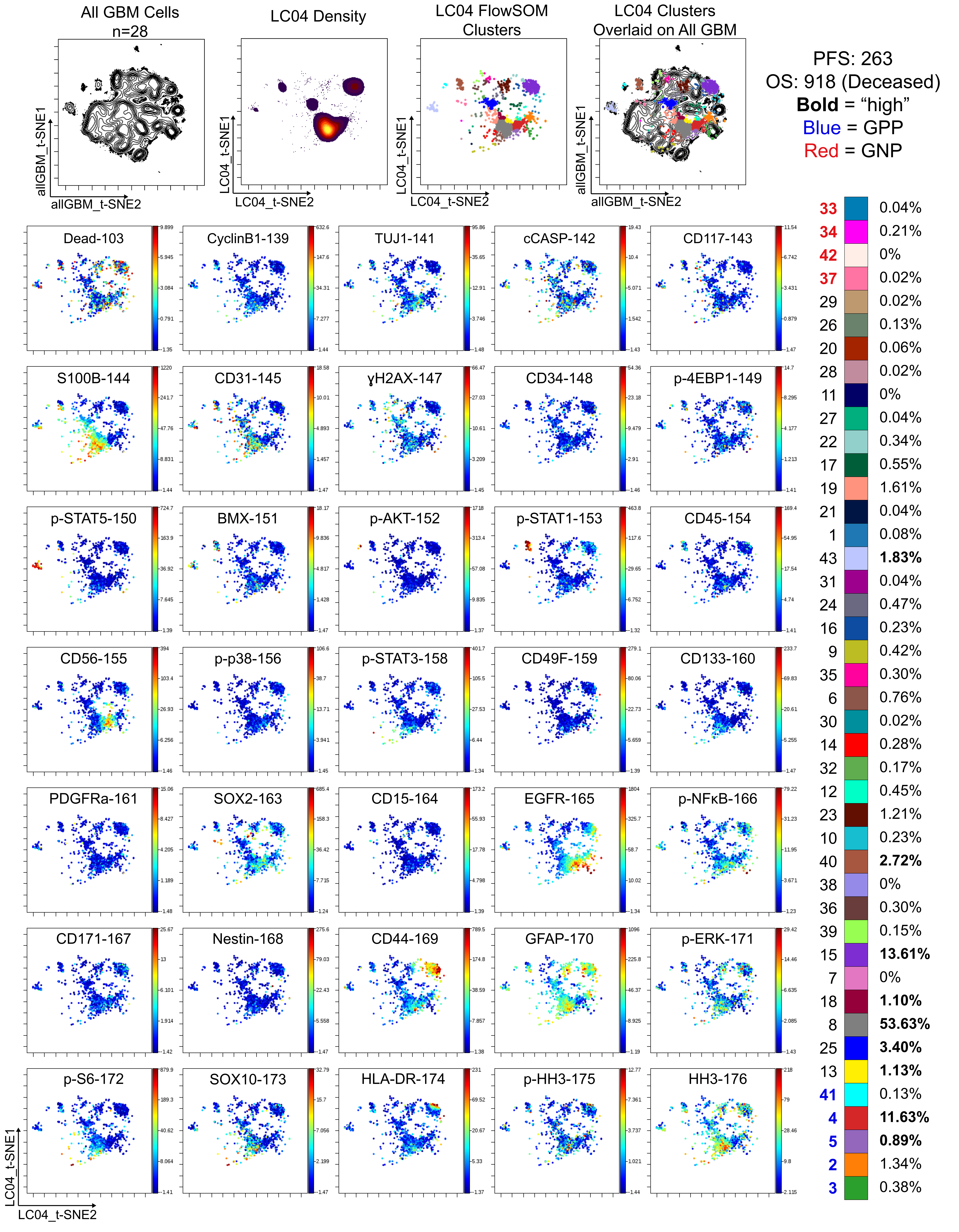

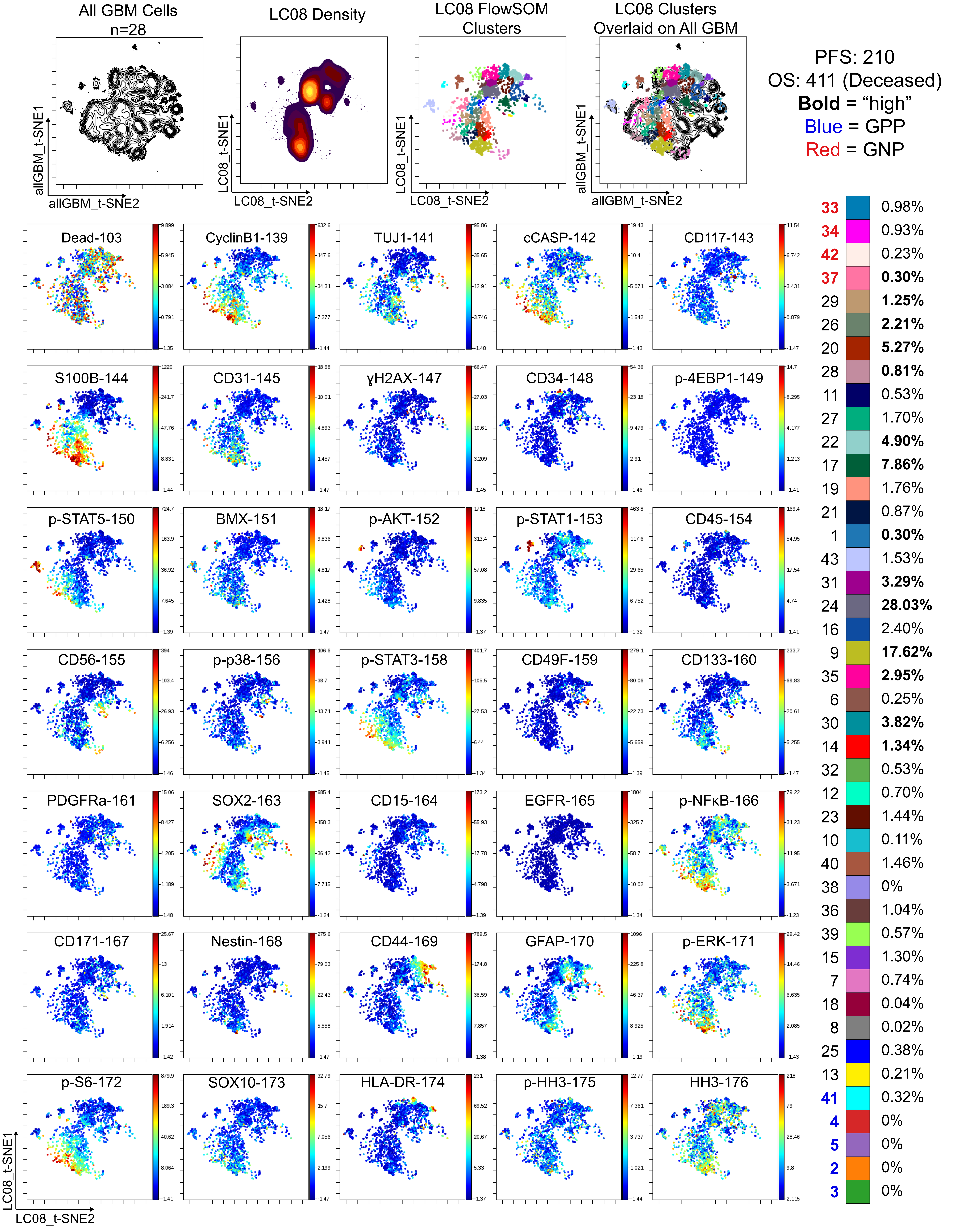
All GBM Cells n=28
LC08 Density
LC08 FlowSOM Clusters
LC08 Clusters Overlaid on All GBM
allGBM_t-SNE1
allGBM_t-SNE2
LC08_t-SNE1
LC08_t-SNE2
PFS: 210
OS: 411 (Deceased)
Bold = "high"
Blue = GPP
Red = GNP
33 0.98%
34 0.93%
42 0.23%
37 0.30%
29 1.25%
26 2.21%
20 5.27%
28 0.81%
11 0.53%
27 1.70%
22 4.90%
17 7.86%
19 1.76%
21 0.87%
1 0.30%
43 1.53%
31 3.29%
24 28.03%
16 2.40%
9 17.62%
35 2.95%
6 0.25%
30 3.82%
14 1.34%
32 0.53%
12 0.70%
23 1.44%
10 0.11%
40 1.46%
38 0%
36 1.04%
39 0.57%
15 1.30%
7 0.74%
18 0.04%
8 0.02%
25 0.38%
13 0.21%
41 0.32%
4 0%
5 0%
2 0%
3 0%
Dead-103
CyclinB1-139
TUJ1-141
cCASP-142
CD117-143
S100B-144
CD31-145
γH2AX-147
CD34-148
p-4EBP1-149
p-STAT5-150
BMX-151
p-AKT-152
p-STAT1-153
CD45-154
CD56-155
p-p38-156
p-STAT3-158
CD49F-159
CD133-160
PDGFRa-161
SOX2-163
CD15-164
EGFR-165
p-NFκB-166
CD171-167
Nestin-168
CD44-169
GFAP-170
p-ERK-171
p-S6-172
SOX10-173
HLA-DR-174
p-HH3-175
HH3-176

Supplemental Single Cell Patient Information

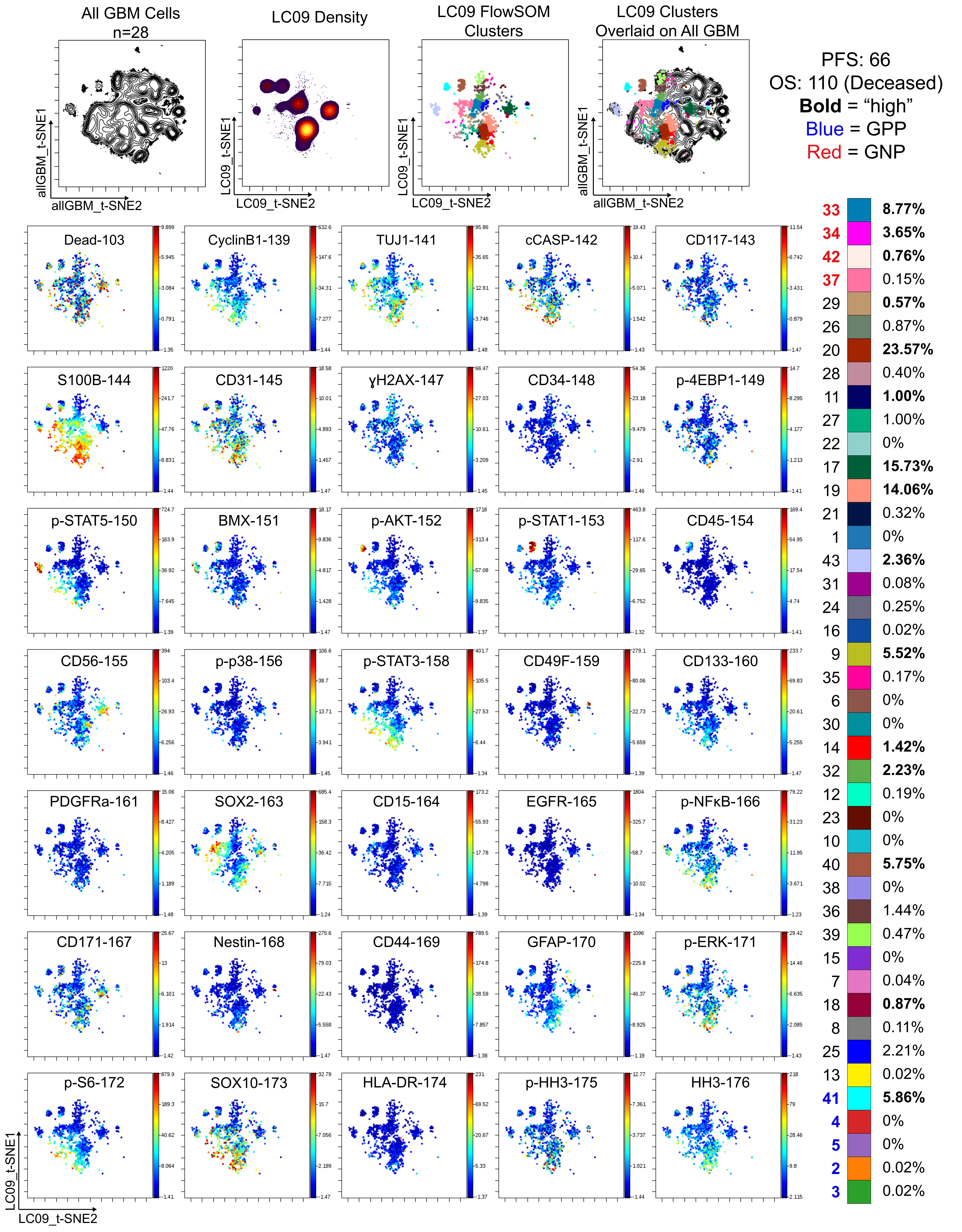

Supplemental Single Cell Patient Information

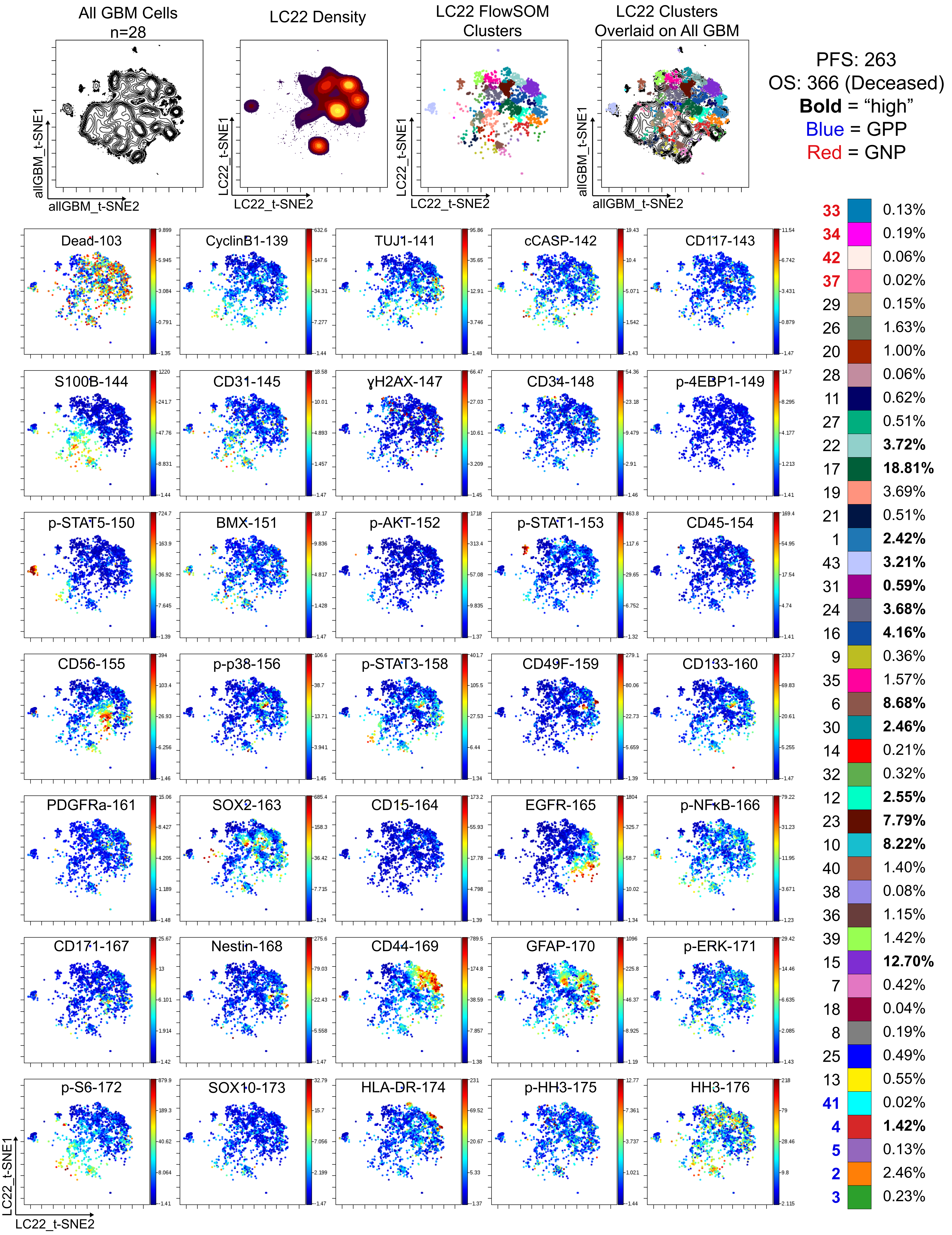

Supplemental Single Cell Patient Information

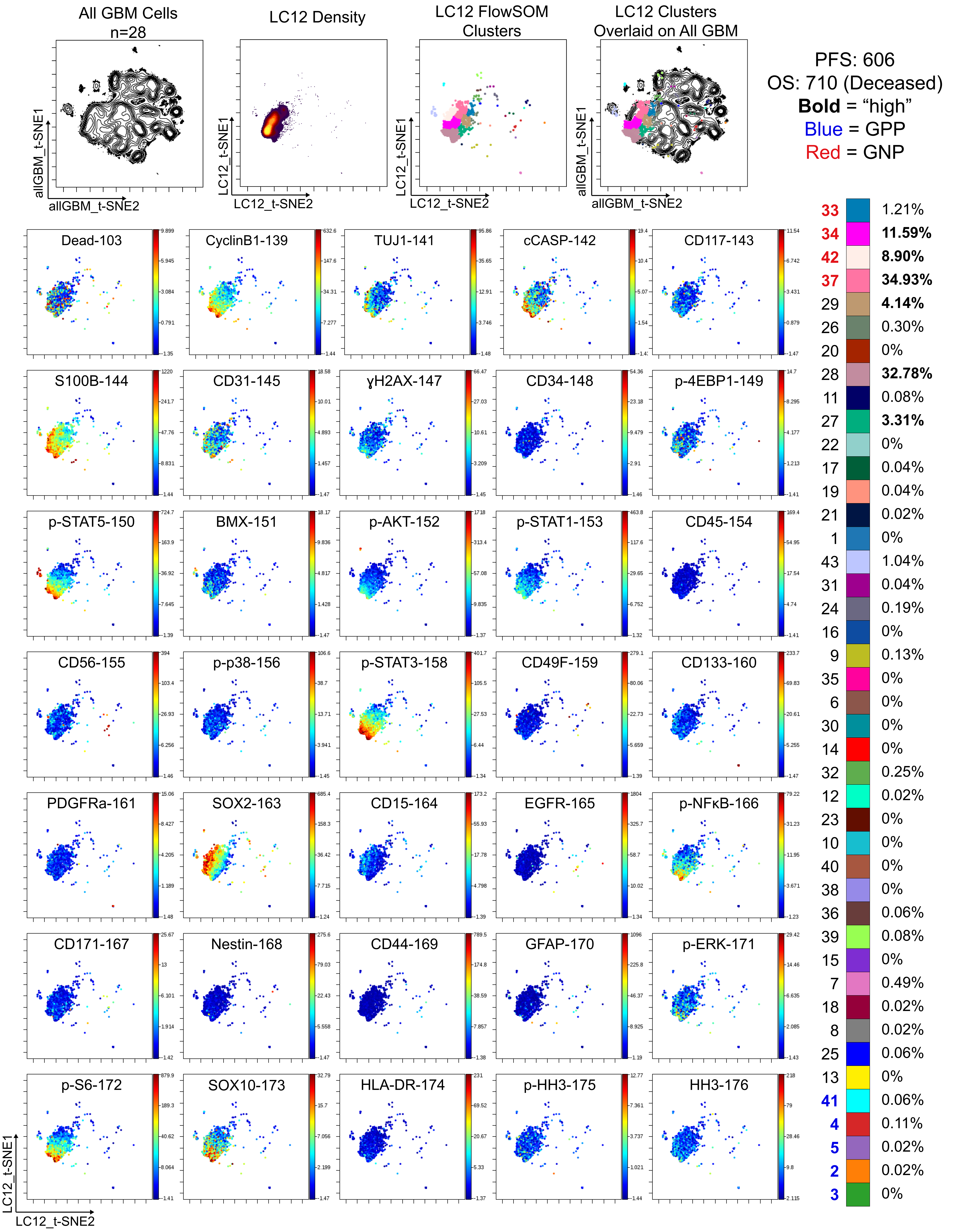

Supplemental Single Cell Patient Information

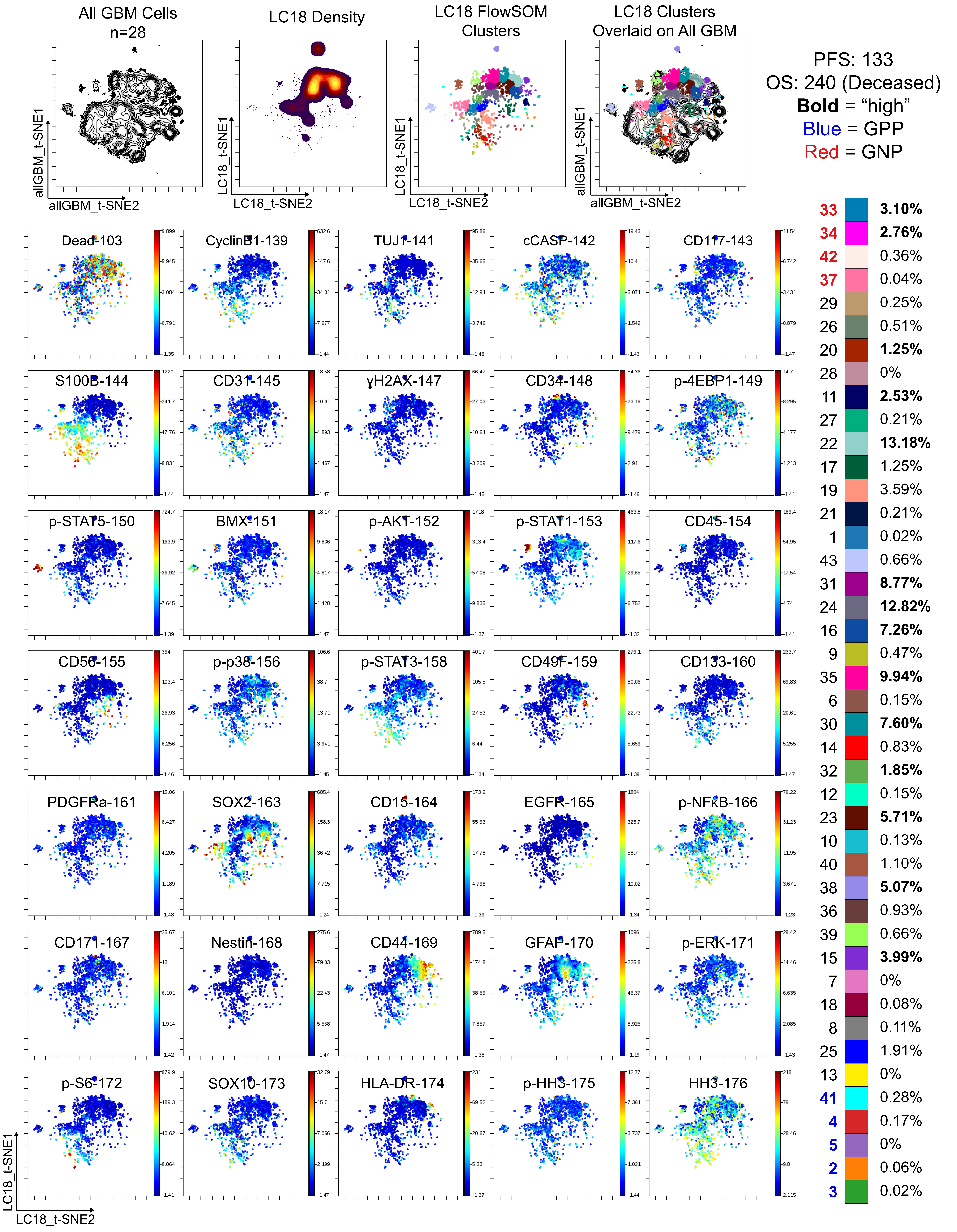

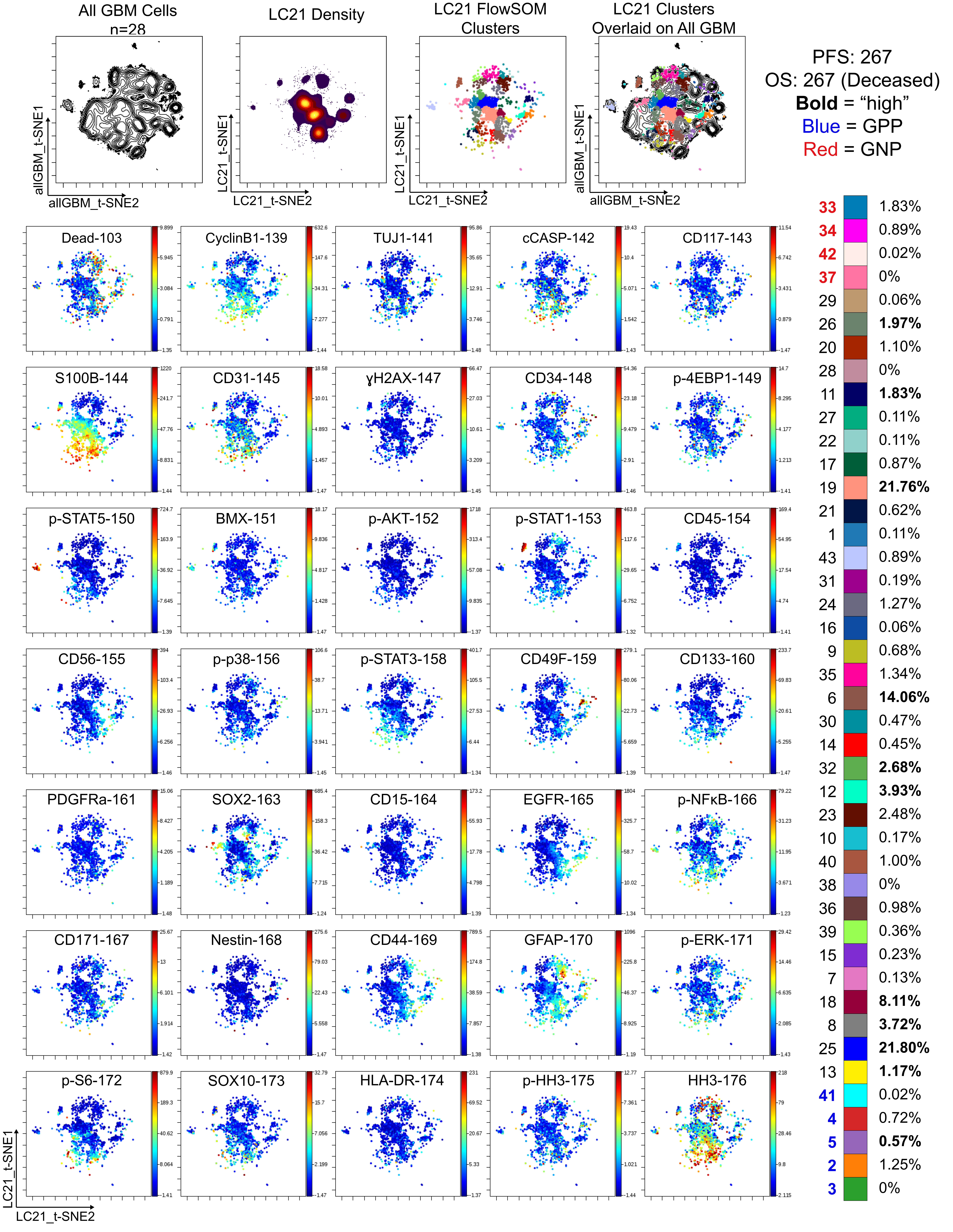
All GBM Cells
n=28
LC21 Density
LC21 FlowSOM Clusters
LC21 Clusters Overlaid on All GBM
allGBM_t-SNE1
allGBM_t-SNE2
LC21_t-SNE1
LC21_t-SNE2
PFS: 267
OS: 267 (Deceased)
Bold = "high"
Blue = GPP
Red = GNP
33 1.83%
34 0.89%
42 0.02%
37 0%
29 0.06%
26 1.97%
20 1.10%
28 0%
11 1.83%
27 0.11%
22 0.11%
17 0.87%
19 21.76%
21 0.62%
1 0.11%
43 0.89%
31 0.19%
24 1.27%
16 0.06%
9 0.68%
35 1.34%
6 14.06%
30 0.47%
14 0.45%
32 2.68%
12 3.93%
23 2.48%
10 0.17%
40 1.00%
38 0%
36 0.98%
39 0.36%
15 0.23%
7 0.13%
18 8.11%
8 3.72%
25 21.80%
13 1.17%
41 0.02%
4 0.72%
5 0.57%
2 1.25%
3 0%
Dead-103
CyclinB1-139
TUJ1-141
cCASP-142
CD117-143
S100B-144
CD31-145
γH2AX-147
CD34-148
p-4EBP1-149
p-STAT5-150
BMX-151
p-AKT-152
p-STAT1-153
CD45-154
CD56-155
p-p38-156
p-STAT3-158
CD49F-159
CD133-160
PDGFRa-161
SOX2-163
CD15-164
EGFR-165
p-NFκB-166
CD171-167
Nestin-168
CD44-169
GFAP-170
p-ERK-171
p-S6-172
SOX10-173
HLA-DR-174
p-HH3-175
HH3-176

Supplemental Single Cell Patient Information

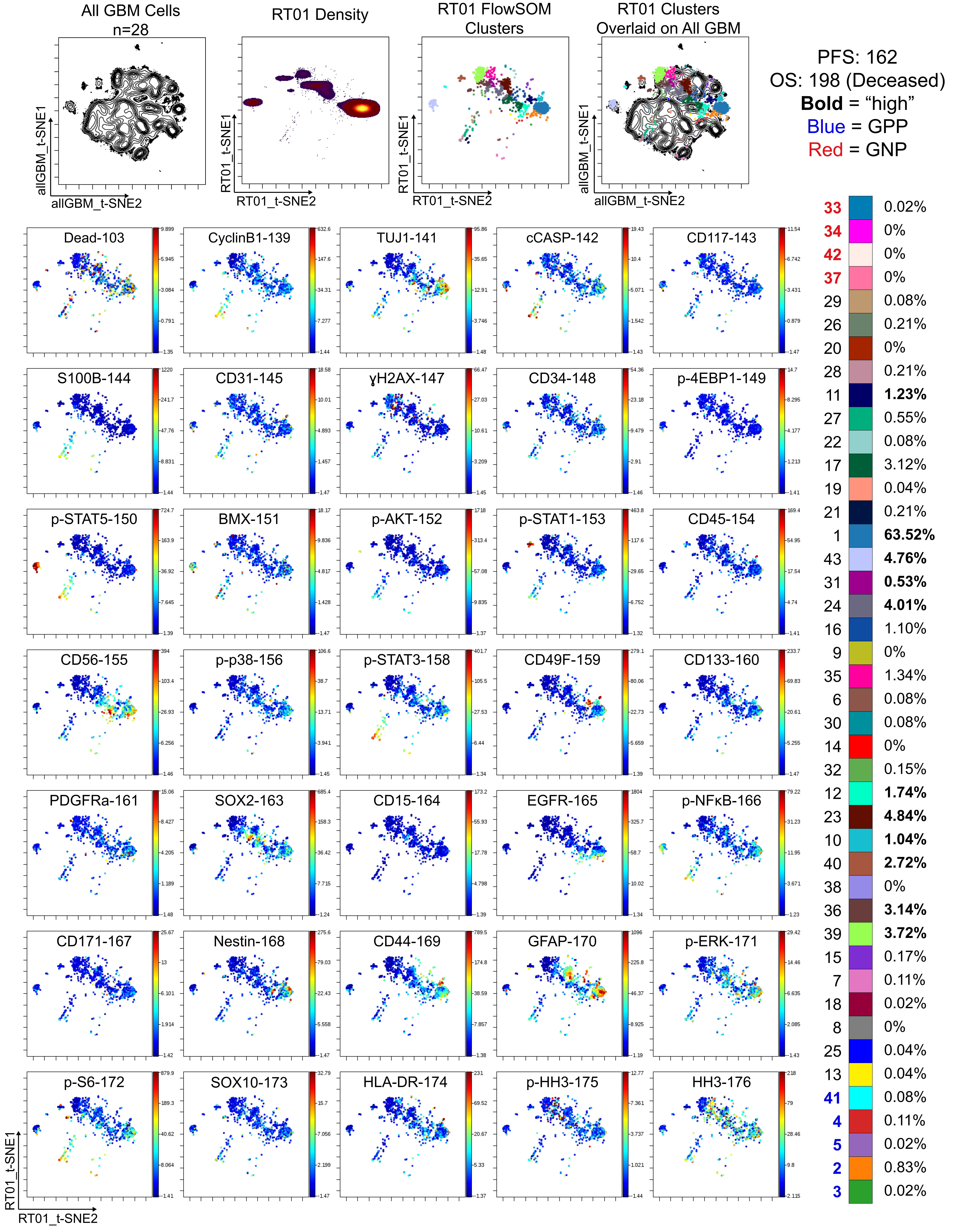

Supplemental Single Cell Patient Information

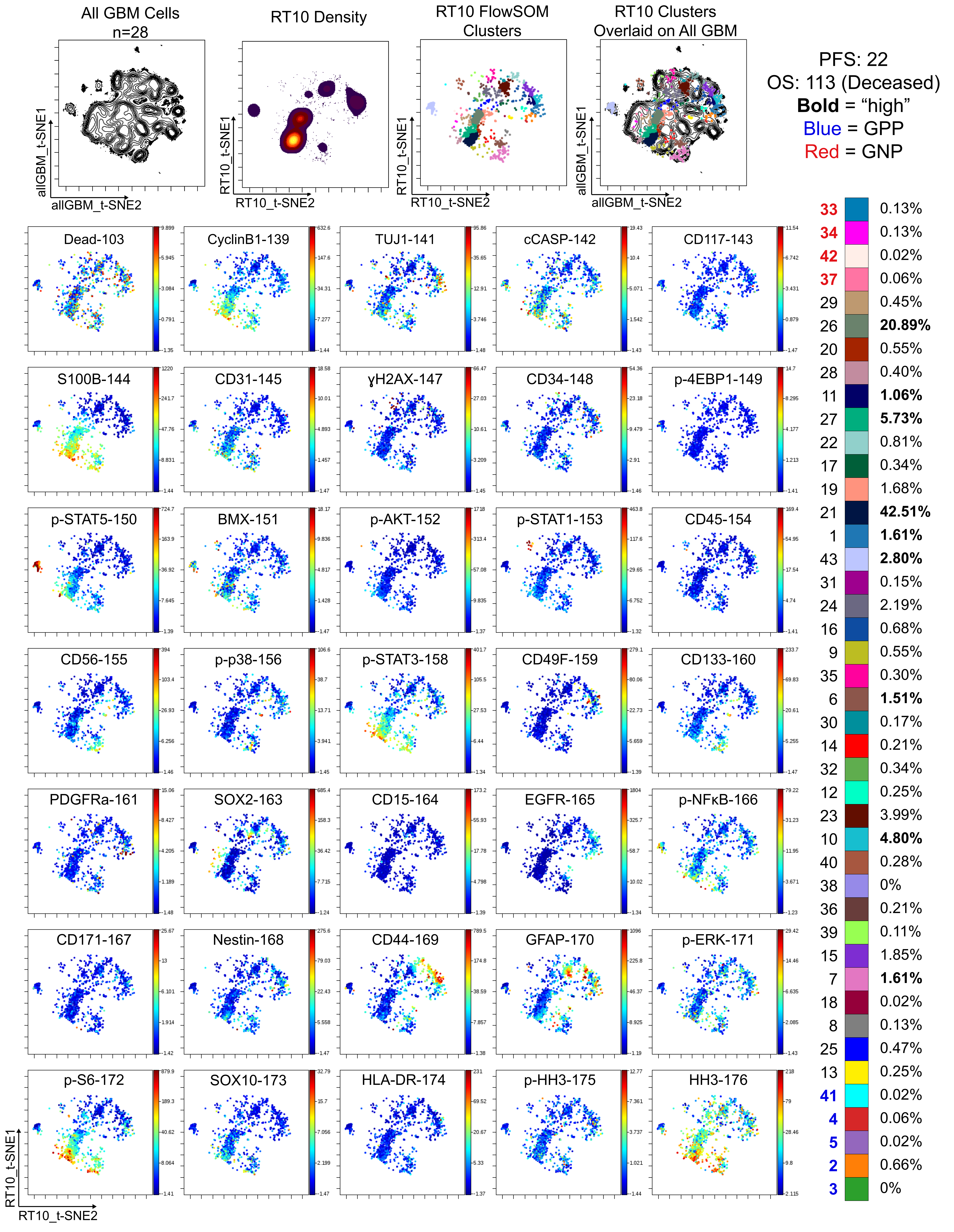

Supplemental Single Cell Patient Information

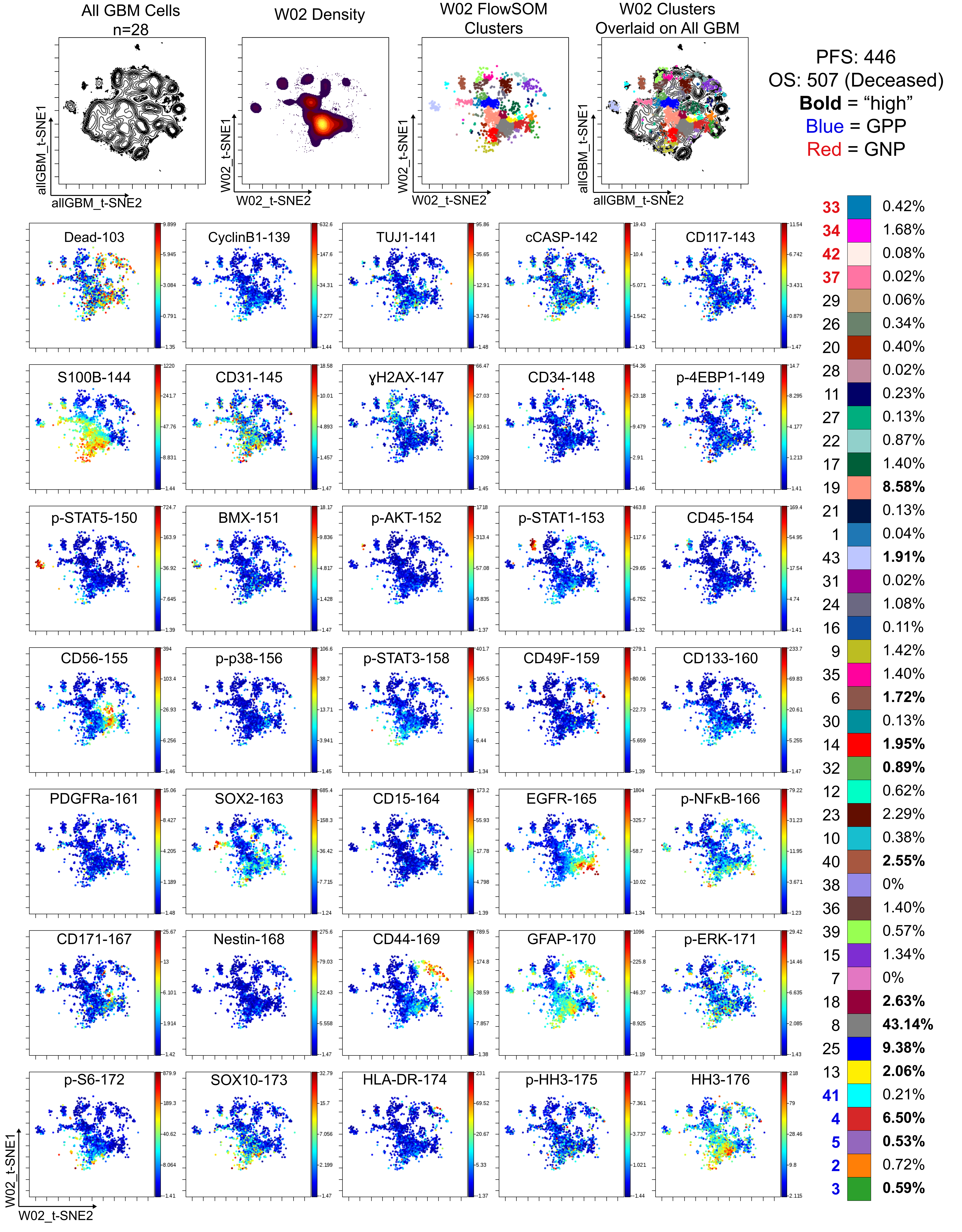

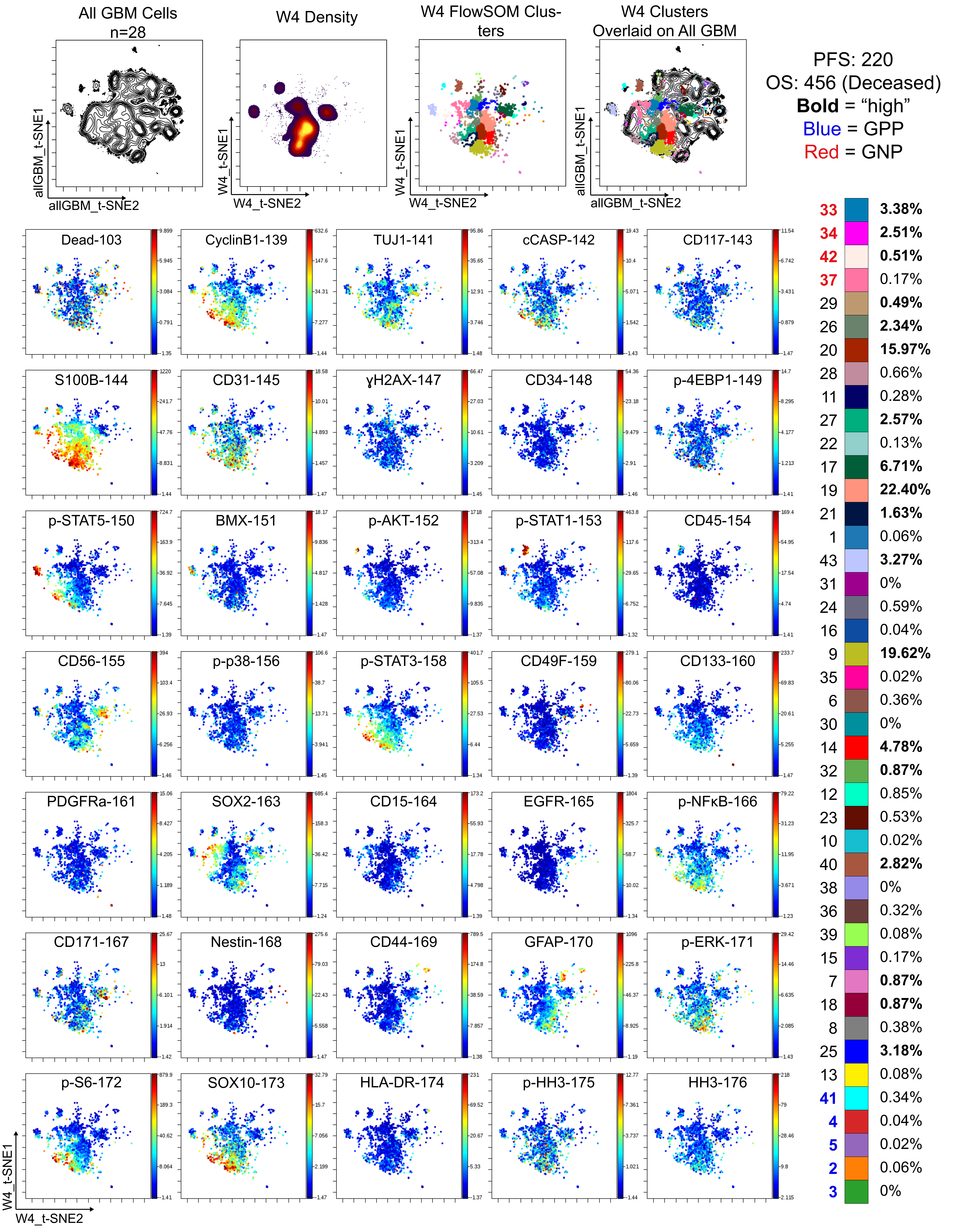

All GBM Cells
n=28
W4 Density
W4 FlowSOM Clus-
ters
W4 Clusters
Overlaid on All GBM
allGBM_t-SNE1
allGBM_t-SNE2
W4_t-SNE1
W4_t-SNE2
PFS: 220
OS: 456 (Deceased)
Bold = "high"
Blue = GPP
Red = GNP
Dead-103
CyclinB1-139
TUJ1-141
cCASP-142
CD117-143
S100B-144
CD31-145
ɣH2AX-147
CD34-148
p-4EBP1-149
p-STAT5-150
BMX-151
p-AKT-152
p-STAT1-153
CD45-154
CD56-155
p-p38-156
p-STAT3-158
CD49F-159
CD133-160
PDGFRa-161
SOX2-163
CD15-164
EGFR-165
p-NFκB-166
CD171-167
Nestin-168
CD44-169
GFAP-170
p-ERK-171
p-S6-172
SOX10-173
HLA-DR-174
p-HH3-175
HH3-176
33 **3.38%**
34 **2.51%**
42 **0.51%**
37 0.17%
29 **0.49%**
26 **2.34%**
20 **15.97%**
28 0.66%
11 0.28%
27 **2.57%**
22 0.13%
17 **6.71%**
19 **22.40%**
21 **1.63%**
1 0.06%
43 **3.27%**
31 0%
24 0.59%
16 0.04%
9 **19.62%**
35 0.02%
6 0.36%
30 0%
14 **4.78%**
32 **0.87%**
12 0.85%
23 0.53%
10 0.02%
40 **2.82%**
38 0%
36 0.32%
39 0.08%
15 0.17%
7 **0.87%**
18 **0.87%**
8 0.38%
25 **3.18%**
13 0.08%
41 0.34%
4 0.04%
5 0.02%
2 0.06%
3 0%

Supplemental Single Cell Patient Information

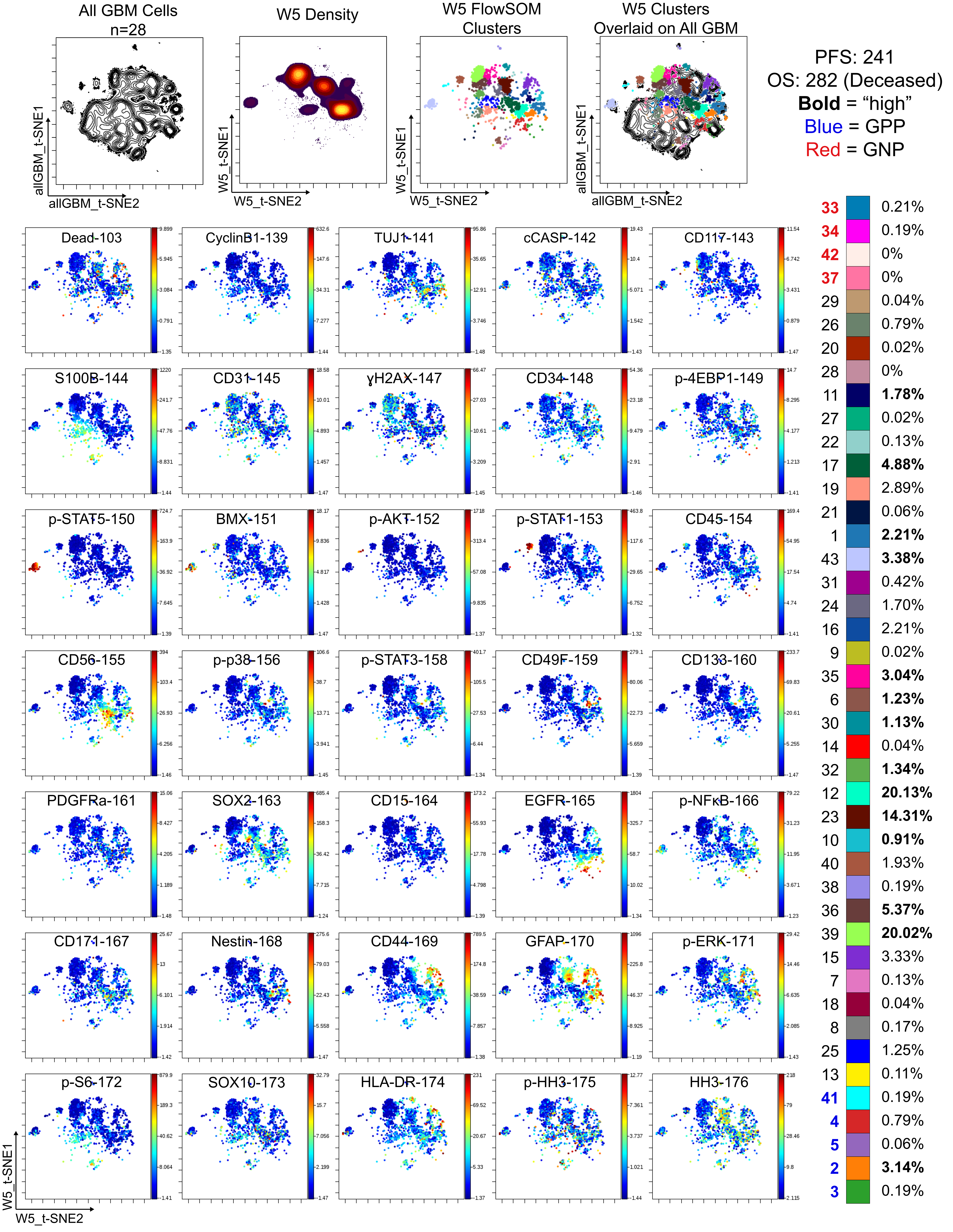

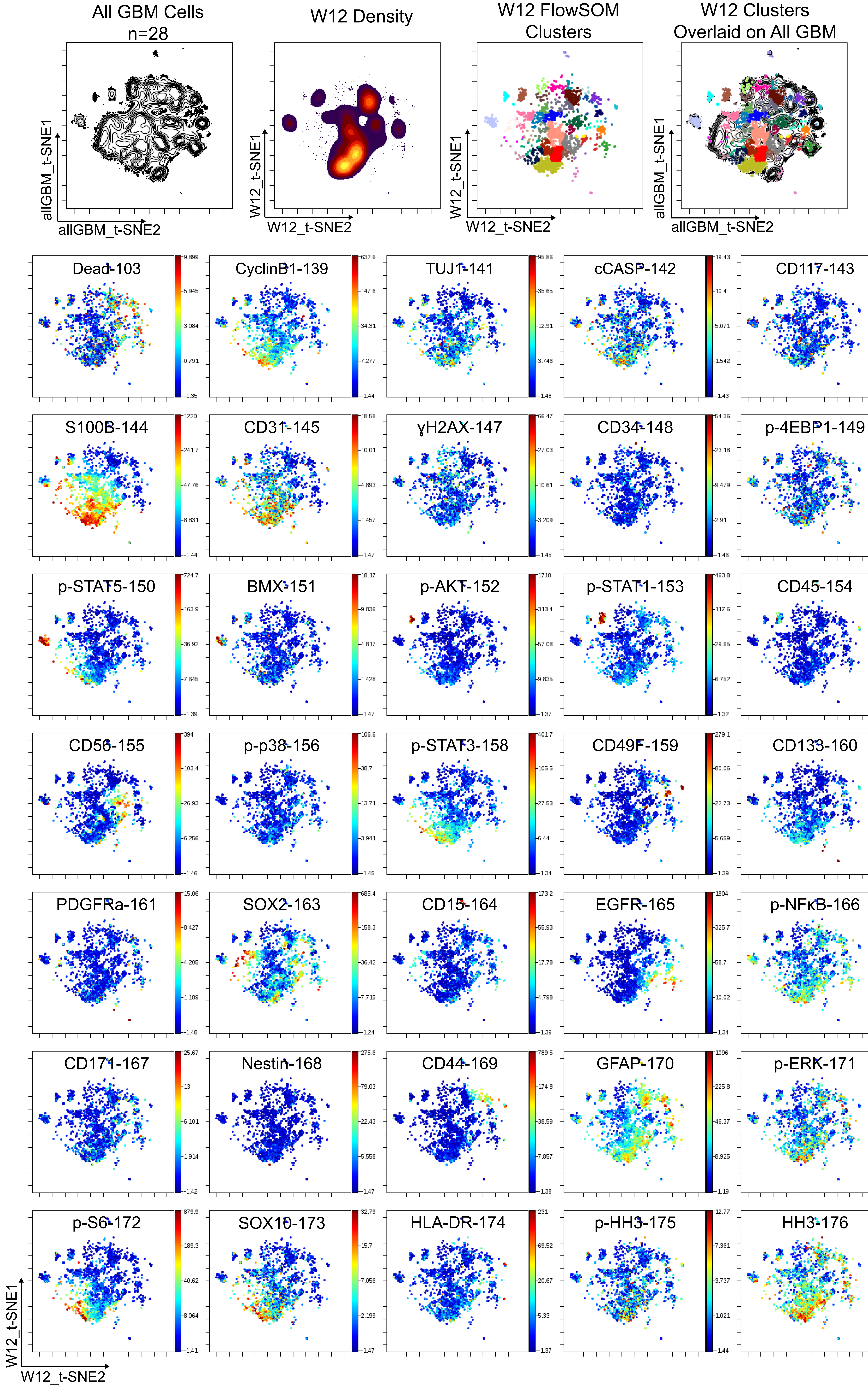

PFS: 190
OS: 723 (Deceased)
Bold = "high"
Blue = GPP
Red = GNP

Cluster	%
**33**	0.98%
**34**	2.08%
**42**	0.28%
**37**	0.08%
29	0.17%
26	0.62%
20	**2.89%**
28	0.23%
11	**0.89%**
27	0.49%
22	0.11%
17	3.65%
19	**9.58%**
21	**1.85%**
1	0.08%
43	**3.04%**
31	0.11%
24	**3.57%**
16	0.64%
9	**22.36%**
35	0.89%
6	**2.91%**
30	0.32%
14	**13.67%**
32	**0.81%**
12	0.32%
23	**9.32%**
10	0.15%
40	**2.34%**
38	0.13%
36	0.49%
39	0.32%
15	1.08%
7	0.28%
18	0.59%
8	**3.74%**
25	**3.01%**
13	0.36%
**41**	**1.08%**
**4**	1.23%
**5**	0.08%
**2**	2.51%
**3**	**0.70%**
