# Supplementary material for: Unsupervised machine learning reveals risk stratifying glioblastoma tumor cells
Source: eLife. 2020 Jun 23;9:e56879. doi: 10.7554/eLife.56879 (PMC7340505; doi:10.7554/eLife.56879)
Supplement: Transparent reporting form [file elife-56879-transrepform.docx]

***eLife’s* transparent reporting form**

**Sample-size estimation**

- You should state whether an appropriate sample size was computed when the study was being designed
- You should state the statistical method of sample size computation and any required assumptions
- If no explicit power analysis was used, you should describe how you decided what sample (replicate) size (number) to use

Please outline where this information can be found within the submission (e.g., sections or figure legends), or explain why this information doesn’t apply to your submission:

For this study, 101 patient samples were analyzed, including 28 patient samples analyzed by mass cytometry and 73 patient samples analyzed in a validation immunohistochemical study. For initial mass cytometry cell subset characterizing, the sample number was chosen based on prior published work using fluorescence or mass cytometry (namely, Irish et al., Cell 2004, Kotecha et al., Cancer Cell 2008, Irish et al., PNAS 2010, Levine et al., Cell 2015, and Good et al., Nature Medicine 2018, all cited in the manuscript). Based on the effect sizes observed in these studies, it was calculated that 25 patients would be required to reveal comparable risk stratifying cell subsets here (N = 28 patients). For the validation cohort (N = 73 patients), the goals were to include all samples where sufficient material and clinical outcome information was available and to at least double the number of distinct patients studied.

**Replicates**

- You should report how often each experiment was performed
- You should include a definition of biological versus technical replication
- The data obtained should be provided and sufficient information should be provided to indicate the number of independent biological and/or technical replicates
- If you encountered any outliers, you should describe how these were handled
- Criteria for exclusion/inclusion of data should be clearly stated

Mass cytometry experiments were performed on each patient sample once. Events identified as live, intact single cells were included in downstream analysis (described in methods). After computational exclusion of immune and endothelial cells, unsupervised analysis of remaining events was performed on a random, equal subsample of the data per-patient for each sample 10 times, confirming that results were consistent across repeated analyses – this testing is reported in the main text (results heading “Statistical validation 2: Clusters identified by RAPID were not dependent on individual patients or sub-samplings” and Figure 3). For imaging data, each sample was represented by multiple cores and final intensity scores represent the median of these cores (described in methods under “Tissue Microarray Construction and Analysis”).

Please outline where this information can be found within the submission (e.g., sections or figure legends), or explain why this information doesn’t apply to your submission:

**Statistical reporting**

- Statistical analysis methods should be described and justified
- Raw data should be presented in figures whenever informative to do so (typically when N per group is less than 10)
- For each experiment, you should identify the statistical tests used, exact values of N, definitions of center, methods of multiple test correction, and dispersion and precision measures (e.g., mean, median, SD, SEM, confidence intervals; and, for the major substantive results, a measure of effect size (e.g., Pearson's r, Cohen's d)
- Report exact p-values wherever possible alongside the summary statistics and 95% confidence intervals. These should be reported for all key questions and not only when the p-value is less than 0.05.

Please outline where this information can be found within the submission (e.g., sections or figure legends), or explain why this information doesn’t apply to your submission:

Statistical testing includes iterative FlowSOM runs, multiple runs of t-SNE, and comparison of subset features across many such runs to identify stable clusters and phenotypes. These statistical validations are noted in subheadings in the results section.

Statistical tests used are extensively described in the methods (under the heading Quantification and Statistical Analysis). Tests reported in each figure are described in the legend of each figure. Additional information (p value, hazard ratio, and IQR for every identified subset) can be found in supplemental table 2.

Because one focus of this paper is the generation of a new analytical method, extensive testing of the robustness of the approach, including application to an independent dataset generated by other researchers, is also included in the manuscript (Figure 2 and 3).

(For large datasets, or papers with a very large number of statistical tests, you may upload a single table file with tests, Ns, etc., with reference to sections in the manuscript.)

**Group allocation**

- Indicate how samples were allocated into experimental groups (in the case of clinical studies, please specify allocation to treatment method); if randomization was used, please also state if restricted randomization was applied
- Indicate if masking was used during group allocation, data collection and/or data analysis

Samples were included only if verified by a pathologist to be *IDH* wild-type glioblastoma and all samples were taken as part of initial surgical resection prior to other treatment. At the time of experimentation, researchers did not have access to outcome data. No further selection was performed prior to analysis and samples; i.e., all samples with enough material for research that were collected within the study time frame were included. The analysis was unsupervised and patient outcome data was not included in the design of the analysis workflow. Patients were not grouped by outcome; rather than look at survival categorically, risk of death was analyzed as a continuous probability using survival in days for groups defined algorithmically as containing a statistically high amount of a phenotypically-distinct cancer cell subset. The algorithm grouped cells into populations in an unsupervised way, and patients were assigned to “high” or “low” cell population groups without any knowledge of, or guidance by, a clinical outcome (Described in the methods under the section “Quantification and Statistical Analysis”). Upon completion of our analysis, it was also confirmed that the cohort of 28 patients analyzed were representative of national demographics of glioblastoma patients (consistent overall and progression free survival trends).

Please outline where this information can be found within the submission (e.g., sections or figure legends), or explain why this information doesn’t apply to your submission:

**Additional data files (“source data”)**

- We encourage you to upload relevant additional data files, such as numerical data that are represented as a graph in a figure, or as a summary table
- Where provided, these should be in the most useful format, and they can be uploaded as “Source data” files linked to a main figure or table
- Include model definition files including the full list of parameters used
- Include code used for data analysis (e.g., R, MatLab)
- Avoid stating that data files are “available upon request”

Please indicate the figures or tables for which source data files have been provided:

Supplemental Table 2 contains numerical data for hazard ratio, F-measures, and p values as well as population abundance and patient outcomes (Figures 1, 3, 4, 5, 6, Figure 1-figure supplements 4-6). Immune cell abundance is also found in Supplemental Table 2. Confidence intervals are reported in figures and as part of the output of RAPID code

TMA Source Data contains numerical data for the dot plot represented in figure 5.

FCS files are available at <https://flowrepository.org/id/FR-FCM-Z24K> (Figures 1, 3-5, and Figure 1 supplements). There are three sets of 28 patient files (Supplementary File 6): 1) Normalized files; 2) Events gated as live, non-immune, non-endothelial cells for each patient; 3) The subsampled events for the t-SNE that was the input to the main text figures.

RAPID Code, scripts, and FCS files from Davis et al. 2018 and the representative t-SNE are available at <https://github.com/cytolab/RAPID>

The Cox proportional-hazards regression model is reported in the methods.
